# Dealing with Discordant Genetic Signal Caused by Hybridisation, Incomplete Lineage Sorting and Paucity of Primary Nucleotide Homologies: A Case Study of Closely Related Members of the Genus *Picris* Subsection *Hieracioides* (Compositae)

**DOI:** 10.1371/journal.pone.0104929

**Published:** 2014-09-05

**Authors:** Marek Slovák, Jaromír Kučera, Eliška Záveská, Peter Vd'ačný

**Affiliations:** 1 Institute of Botany, Slovak Academy of Sciences, Bratislava, Slovakia; 2 Department of Botany, Charles University, Praha, Czech Republic; 3 Department of Zoology, Comenius University, Bratislava, Slovakia; Montreal Botanical Garden, Canada

## Abstract

We investigated genetic variation and evolutionary history of closely related taxa of *Picris* subsect. *Hieracioides* with major focus on the widely distributed *P. hieracioides* and its closely related congeners, *P. hispidissima*, *P. japonica*, *P. olympica*, and *P. nuristanica*. Accessions from 140 sample sites of the investigated *Picris* taxa were analyzed on the infra- and the inter-specific level using nuclear (ITS1-5.8S-ITS2 region) and chloroplast (*rpl32*-*trnL* ^(UAG)^ region) DNA sequences. Genetic patterns of *P. hieracioides*, *P. hispidissima*, and *P. olympica* were shown to be incongruent and, in several cases, both plastid and nuclear alleles transcended borders of the taxa and genetic lineages. The widespread *P. hieracioides* was genetically highly variable and non-monophyletic across both markers, with allele groups having particular geographic distributions. Generally, all gene trees and networks displayed only a limited and statistically rather unsupported resolution among ingroup taxa causing their phylogenetic relationships to remain rather unresolved. More light on these intricate evolutionary relationships was cast by the Bayesian coalescent-based analysis, although some relationships were still left unresolved. A combination of suite of phylogenetic analyses revealed the ingroup taxa to represent a complex of genetically closely related and morphologically similar entities that have undergone a highly dynamic and recent evolution. This has been especially affected by the extensive and recurrent gene flow among and within the studied taxa and/or by the maintenance of ancestral variation. Paucity of phylogenetically informative signal further hampers the reconstruction of relationships on the infra- as well as on the inter-specific level. In the present study, we have demonstrated that a combination of various phylogenetic analyses of datasets with extremely complex and incongruent phylogenetic signal may shed more light on the interrelationships and evolutionary history of analysed species groups.

## Introduction

Identifying patterns of genetic variation within and among closely allied congeners is crucial in understanding their evolutionary relationships and processes involved in their diversification. Exploration of genetic patterns across the entire geographic ranges of each of the constituent species not only enables clarification of modes of their diversification and historical changes in spatial distribution, but may also shed light on their potential reciprocal interactions (e.g. hybridisation and introgression).

In many instances, however, multi-gene phylogenies of closely related and recently diversified taxa are incongruent, often with alleles transcending species or genetic groups, and finally cause the genetic patterns to exhibit a diffusive or mosaic-like structure [1], [2], [3], [4], [5]. A suite of various factors and processes might lead to discordant topologies of genetic trees and complex genetic patterns. The most commonly detected evolutionary events affecting genetic variation are hybridization and introgression, gene duplication, incomplete lineage sorting and/or recombination [6], [7], [8], [9]. Eventually, the low level of variation in the involved genetic markers and the different molecular nature of employed coding and non-coding regions in nuclear, mitochondrial and chloroplast DNA may be a source of gene trees incongruence. Subjective taxonomic delimitation of studied taxa not corresponding with real evolutionary patterns must be also taken into account [10], [11]. It may prove difficult to correctly diagnose the relative influence of those factors on the studied species, because they all result in very similar complex genetic patterns and discordant phylogenetic inferences [9], [12], [13].

Studies that have carefully and explicitly analyzed multiple reasons of genetic incongruence are not common [14], [15], [16], [17]. On the contrary, the overwhelming majority of studies were focused on only a subset of possible reasons of incongruence, however, using a variety of different approaches [1], [2], [3], [5]. Reconstruction of species relationships, i.e. species trees based on multiple gene trees, is recently performed using coalescent models that can handle incongruence caused by incomplete lineage sorting [18]. However, these models are applicable only to datasets where no gene flow (hybridization) among species is to be expected [19], [20]. Indeed, it is fairly difficult to distinguish between effects caused by hybridisation and incomplete lineage sorting [9]. One way how to overcome these difficulties has been proposed by Joly et al. [9] who developed an approach enabling to test the influence of hybridization in the presence of incomplete lineage sorting, as implemented in the software JML [20].

Members of *Picris* subsect. *Hieracioides* Vassiliev (Compositae) [21] represent an interesting group for investigation of genetic patterns, diversification and reciprocal interactions within and among closely related species that differ in ecological requirements and geographic distribution [22], [23], [24]. According to the current taxonomic concepts [21], [23], [24], *Picris* subsect. *Hieracioides* comprises seven morphologically very similar taxa from Europe, Asia and Africa: *P. abyssinica* Sch. Bip., *P. hieracioides* L. including subspecies *P. hieracioides* subsp. *umbellata* (Schrank) Ces. and *P. hieracioides* L. subsp. *hieracioides* (referred as *P. h.* subsp. *umbellata* and *P. h.* subsp. *hieracioides* in the text), *P. hispidissima* (Bartl.) W. D. J. Koch, *P. japonica* Thunb., *P. nuristanica* Bornm., and *P. olympica* Boiss [21], [24], [25]. Information on overall relationships within the genus *Picris* subsect. *Hieracioides* is still limited. Only three taxa from the subsection *Hieracioides*, namely *P. abyssinica*, *P. hieracioides* and *P. nuristanica*, were marginally analysed in a study focused on the genus *Leontodon*. The two latter *Picris* species were shown to be closely related, while *P. abyssinca* was genetically much more distant and clustered together with other North African *Picris* taxa [26]. All members of the subsection *Hieracioides* are reported to be perennials with homocarpic achenes (i.e. with same morphological type of achenes within capitulum) and two-hooked anchor hairs on the stem and peduncles [21]. Here, we focus on species from Europe and Asia Minor, with main attention paid besides of the widespread and highly variable *P. hieracioides* also to *P. hispidissima*, *P. olympica* and marginally also to *P. japonica* and *P. nuristanica*.

Although our previous investigations on *P. hieracioides*, *P. hispidissima*, *P. japonica*, and *P. nuristanica* revealed that these taxa are very similar morphologically and karyologically as well as they are closely related genetically (AFLP), their evolutionary relationships and mutual interactions were left unresolved [22]–[24], [27], [28]. Additionally, our previous studies [23], [24] did detect morphologically intermediate plants in mixed populations of *P*. *hispidissima* and *P. hieracioides* and also found populations that represented genetic admixtures of different *P. hieracioides* subspecies and/or genetic groups, mostly accompanied by transitional morphology. In all cases, the observed intermediate genetic and/or morphological states were hypothetically attributed to hybridisation although this heterogeneity may be also a result of retention of ancestral variation. No studies collecting the relevant data on *P. olympica* have been done prior to the present study. Likewise, overall relationships within *Picris* subsect. *Hieracioides* have not been investigated yet. Thus the specific goals of the present study are:

1. Clarifying the phylogenetic relationships among *P. hieracioides, P. hispidissima, P. japonica, P. olympica, and P. nuristanica*.
2. Exploring the overall genetic pattern of *P. hieracioides* in Europe and Asia Minor, the distribution of its infraspecific genetic groups, and comparing its genetic variation with that of *P. hispidissima* and *P. olympica*.
3. Shed more light on potential interactions within the studied group and on the processes acting during evolutionary history.

## Material and Methods

### Studied species

*Picris hieracioides* is a biennial to perennial, morphologically highly variable species that includes two infraspecific taxa, *P. h.* subsp. *umbellata* and *P. h.* subsp. *hieracioides*. The subspecies of *P. hieracioides* are morphologically distinguishable by a combination of several morphological traits on both vegetative and reproductive organs. This was definitely proved also by cultivation experiments under environmentally homogeneous conditions [24]. Beside this, they also differ in longevity, ecological requirements, and geographic distribution [22], [24]. Additionally, each subspecies harbours two highly allopatric genetic (AFLP) and morphologically cryptic groups [24]. *Picris hispidissima* is a biennial species (M. Slovák, unpublished data) that differs morphologically from *P. hieracioides* in having a pectinate-ciliate indumentum of involucral bracts and inflation of the peduncle [23]. The last studied species, *P. olympica*, is a perennial characterized by a low caespitose growth with quite short ascending scapose-like stems bearing only a few capitula. It is considered to be morphologically closely related to *P. hieracioides* [21], [29]. The investigated species differ conspicuously in their ecological requirements and geographic ranges, with *P. hieracioides* being the most widespread (distributed across the major part of Europe and Asia Minor) and having wide ecological amplitude [21], [24]. The nominate *P. h.* subs. *hieracioides* is mainly biennial, growing on open, dry, xerothermous biotopes with no bedrock preference and thriving in lowlands or lower mountains throughout all of Europe. In contrast, *P. h.* subsp. *umbellata* is a short-lived perennial herb inhabiting humid tall herb communities from montane forests to sub-alpine communities across European calcareous mountain ranges. Both *P. hispidissima* and *P. olympica* have restricted distributions. The former occurs in dry, calcareous rocky slopes and crevices in the Adriatic coastal mountains from Croatia to Montenegro, while the latter is restricted to a few high mountain ranges in western Anatolia where it inhabits open, craggy grasslands with alkaline bedrocks at alpine levels [21], [29]. All species, but especially *P. hieracioides*, have spread from their natural biotopes into man-made habitats and migrated along anthropogenic corridors ([22], [24]; M. Slovák unpublished data).

### Sampling strategy and plant material

In order to perform the reconstruction of phylogenetic relationships among the studied species and to reveal their genetic patterns, we aimed to include as many populations as possible from the entire geographic ranges of *P. hieracioides*, *P. hispidissima*, and *P. olympica* (Table S1). This, however, was at the expense of sample size at the population level [30], [31], [32]. We also co-analyzed a limited number of samples of the closely related Asian taxa *P. japonica* (2 populations) and *P. nuristanica* (1 population). We did not include samples of *P. abyssinica* since it was proved that it is genetically highly divergent from *P. hieracioides* and *P. nuristanica* but is more closely related to other African taxa [26]. Each population of the investigated species had more than several hundreds of individuals, and the taxa under study are neither endangered nor protected and no specific permits were required to collect plant samples at the study sites. We sequenced one to three accessions per population for the nuclear ITS and the plastid *rpl32-trnL* ^(UAG)^ regions (Table S1). To cover as high proportion of genetic variation of analysed taxa as possible, but simultaneously with respect to sampling limitation at population level, we decided to analyse more than one individual for populations where non-homogenized ITS sequences indicated existence of more than a single allele. The widespread *P. hieracioides* including populations morphologically corresponding to both subspecies, *P. h.* subsp. *hieracioides* and *P. h.* subsp. *umbellata*, was collected from 122 sample sites covering both subspecies' entire geographic distributions, and henceforth these sites are referred to as populations (Table S1). Twelve populations of *P. hispidissima* from its entire distribution area were sampled [23]. Three of the five currently recognized populations of *P. olympica* were also collected for the purposes of the present study.

Intermediate morphological variation was identified in a few populations of *P. hieracioides* which were mostly located in zones where the two subspecies or their lineages meet [24]. Since the extent of intermediate morphological variation across these populations was wide, it was impossible to unequivocally sort some of them into discrete categories. Therefore, in order to avoid formation of artificial entities, which might lead to subsequent biasness of results and interpretations, we assigned particular populations a priori not to accepted subspecies, but we considered all of them as *P. hieracioides* instead.

Selection of appropriate outgroup taxa was based on previously published studies [21], [26] and our preliminary phylogenetic analyses. Thus a priori selected outgroup comprised members of all sections of the genus *Picris* (*P. galilaea* (Boiss.) Eig., *P. capuligera* (Durieu) Walp., *P. pauciflora* Willd., *P. rhagodioloides* (L.) Desf., *P. scaberrima* Ten., *P. sinuata* (Lam.) Lack, and *P. strigosa* M. Bieb.) and two members of the closely related genus *Helminthotheca* (*H. aculeata* (Vahl) Lack and *H. echioides* (L.) Holub). Voucher specimens were deposited in the herbarium of the Institute of Botany at the Slovak Academy of Science (SAV).

### DNA sequence markers, DNA extraction and PCR amplification

We utilised sequences of the nuclear ribosomal internal transcribed spacer (ITS1-5.8S-ITS2 region) and the non-coding cpDNA intergenic spacer *rpl32*-*trnL* ^(UAG)^ [33]. Several single-copy nuclear genes (A25, A28 and B12) [34] and another non-coding chloroplast regions (3′*trn*V^(UAC)^-*ndh*C, *ndhF-rpl32* and *trn*H^(GUG)^-*psb*A) [33], [35] were tested during preliminary screening. However, these were proved to be unsuitable for the present study due to low levels of variation and/or problems encountered during their PCR amplification.

Total genomic DNA was extracted from silica gel-dried leaf tissue by the DNeasy plant mini kit (Qiagen, Hilden, Germany), following the manufacturer's protocol. The ITS region and the plastid *rpl32*-*trnL* ^(UAG)^ spacer (referred as cpDNA henceforth in the text) were amplified using the standard PCR reaction, employing the following universal primer pairs: P1A, P4 and internal ones, ITS2 and ITS3, if necessary [36], [37] and *trnL-retF* and *rpl32-retR* [39].

Amplifications were carried out in a Mastercycler ep Gradient S thermal cycler (Eppendorf, Hamburg, Germany), using PCR reaction volumes of 25 µL consisting of 0.75 U of *Pfu* polymerase (Fermentas, St. Leon-Rot, Germany), 0.2 mM of each dNTP, 0.2 µM of each primer, 1 µL of DNA template, and reaction buffer containing 2 mM of MgSO_4_ (Fermentas, St. Leon-Rot, Germany). For ITS amplification, we used the following thermocycler program: preheating at 94°C for 3 min, then running it for 35 cycles at 94°C for 30 sec, then at 50°C for 30 sec, and 72°C for 60 sec, with the final extension at 72°C for 10 min, and cooling at 4°C. The cpDNA region was amplified using the “*rpl16*” program, as described by [38]. PCR products were purified using the NucleoSpin Extract II kit (Macherey-Nagel, Düren, Germany). Cycle sequencing reactions were carried out using the same primers, and sequencing was performed on an ABI PRISM 3130*xl* sequencer at BITCET Consortium, Comenius University in Bratislava. The sequencing of a few accessions repeatedly failed for the ITS region and these were therefore excluded from further analyses (Table S1).

### Sequence alignments and phylogenetic analysis

Sequences were edited and aligned manually using the BioEdit program version 7.0.4.1 [39]. In case of ITS sequences, the electroferograms were carefully inspected for intra-individual polymorphic sites (IPS) having more than one signal (cf [40]). These were labelled with NC-IUPAC ambiguity codes. Polymorphic positions within ITS sequences, in which both bases were detected also separately in different accessions elsewhere in the alignment, were considered additive polymorphic sites (APS) [40].

We believe that the observed intra-individual variation did not arise from PCR errors, because numerous accessions of *P. hieracioides* and also other taxa possessed homogenized ITS sequences. The geographic pattern of homogeneous and heterogeneous ITS sequences was obviously not random, indicating a genuine variation rather than erroneous signals caused by inaccurate amplification. Although the intra-individual ITS variation might be revealed by cloning procedure, this approach, on the other hand, may result in amplification of numerous ITS sequence types which do not represent relevant variability [41]. Incomplete concerted evolution and/or recombination, processes operating on the multiple-copy regions like ITS, increase the number of unwanted sequence types making the ITS phylogenetic analysis even more complex. Furthermore, with our current sampling involving hundreds of accessions exhibiting intra-individual ITS polymorphism, the cloning procedure would be out of the reasonable solution. As long as most of the sequences were possible to obtain by direct sequencing and polymorphic sites could be designated by IUPAC ambiguity codes, we decided to separately analyse two ITS datasets, one including and one excluding the individuals with APS (see below).

Altogether we generated three alignments: the first one was designated as ITS_1 and involved 117 sequences without APS (Alignment S1. ITS_1). The second one was designated as ITS_2 and comprised 216 sequences coming from all accessions analysed for the ITS region and including all sequences with APS (Alignment S2. ITS_2). The second alignment was constructed after considering that the intra-individual polymorphic sites, especially those with APS, may significantly influence the hierarchical structure of the phylogenetic trees.

Finally, the third cpDNA alignment comprised all plastid sequences, in particular 219 accessions (Alignment S3. cpDNA). In order to detect possible incongruence between particular markers, the incongruence length difference (ILD) test [42], as implemented in the “partition homogeneity” test of PAUP* (1000 replicates, Mulpars off, outgroups included), was employed. Although topologies of the ITS and cpDNA trees were shown to be incongruent, statistical support especially at their basal nodes was comparatively poor, causing their main branching patterns to be unsupported. Therefore, we decided to take the advantage of synergistic effect of combining datasets and generated also concatenated alignments. The concatenated alignment, ITS_cpDNA, contained 117 ITS sequences without APS and corresponding cpDNA sequences (Alignment S4. ITS_cpDNA).

Indels were not coded separately but treated as missing data. The aligned datasets were analysed independently using the following phylogenetic approaches:

1. Maximum parsimony (MP) phylogenetic analysis was performed with the heuristic search option in PAUP* version 4.0b10 [43]. The following settings were utilised: accelerated character transformation (ACCTRAN), gaps treated as missing data, single-site polymorphisms determined uncertainties, tree construction with stepwise addition, 1000 bootstrap replicates with random taxon addition, tree bisection-reconnection (TBR) branch swapping, and retention of multiple trees found during branch swapping (MULTREES option in effect). The identical sequences were merged in McClade version 4.0 PPC [44] to reduce computation time. Clade support was calculated via bootstrap analyses using 10000 re-samplings done with the fast heuristic search in PAUP*. Bootstrap support was categorized according to the following criteria: strong (>85%), moderate (70%–85%), weak (50%–69%), or poor (<50%).
2. Bayesian inference (BI) was run in MrBayes version 3.1.2, using the Markov Chain Monte Carlo algorithm (MCMC) [45]. Bayesian analyses were performed on the CIPRES Portal version 1.15 [46]. Prior to Bayesian analyses, the most appropriate nucleotide substitution models were chosen, using the Akaike Information Criterion (AIC) in jMODELTEST version 0.0.1 [47], [48]. Evolutionary models were calculated for each part of the datasets separately. Specifically, the ITS datasets contained partitions corresponding to the ITS1 and ITS2 spacers and the 5.8S rRNA gene, while concatenated datasets included besides the three ITS region partitions also a fourth partition represented by the cpDNA region. The following models or model combinations were found to be the most appropriate for the datasets studied: (1) ITS_1 and ITS_2 datasets – the SYM + G model for ITS1 and ITS2 sequences and the K80 model for 5.8S rRNA gene sequences; (2) cpDNA dataset – the TVM + I + G model; (3) concatenated ITS_cpDNA – the SYM + G model for ITS1 and ITS2, the K80 model for the 5.8S rRNA gene sequences, and the TVM + I + G model for the cpDNA partition.

All BI analyses were run with four independent Metropolis-coupled MCMC chains (three heated and one cold chain) for ten to twenty five million generations and sampled every 1000^th^ generation. The first 25% of sampled trees were regarded as ‘burn-in’ trees and were discarded prior to reconstruction of a 50% majority-rule consensus tree. Stationarity was confirmed by checking convergence diagnostic parameters. Specifically, the average standard deviation of split frequencies was lower than 0.01 in all cases; the plots of generations versus log probability of the observed data showed no obvious trends; and the Potential Scale Reduction Factor (PSRF) approached 1. Finally, topologies and node posterior probability values were compared among the runs. The topologies were stabilized among all datasets with only minute differences in branching pattern of terminal clades. Nodes with posterior probability (PP) values of 0.90 and above were regarded as significant and those with PP values below 0.90 regarded as non-significant.

3. Net-like approaches were used to identify and display potential contradictory signals in the datasets. All alignments including ITS_2 containing intra-individual polymorphic sites were analysed using the neighbour-net analysis of [49] in SplitsTree version 4.10 with uncorrected P-distance and default settings. To visualize the relationships among cpDNA haplotypes and to detect possible ancestral polymorphism, the cpDNA dataset was subjected to haplotype network analysis based on the parsimony method of [50] using TCS version 1.21 [51] limited to 30 steps of parsimonious connection in creating the network.
4. In order to precisely specify the amount of phylogenetically informative signal in the datasets, we analysed spectrum of supporting nucleotide positions. There are three groups of supporting positions recognized by Wägelle and Rödding [52]: (1) symmetrical or binary positions have two different character states in functional outgroup and ingroup and thus support both group of a split equally; (2) asymmetrical positions support only one group which possesses the same nucleotide at particular position, while the other group harbours different and more than one character state at this position; (3) noisy positions include same character states present in all sequences of the functional ingroup but also at least in one sequence of the functional out-group and thus represent convergences or chance similarities between ingroup and outgroup, or alternatively ingroup autapomorphies.
5. We also apply a Bayesian coalescent-based approach to estimate a species tree employing *BEAST as implemented in the program BEAST version 1.7.4 [18]. Two input files, one with 117 homogeneous ITS sequences (Alignment S1. ITS_1) and the second one containing corresponding cpDNA sequences, were used for the BEAST analysis. Populations assigned to particular taxa were used as OTU's. The input file for *BEAST was created in BEAUti version 1.7.4, with the following settings: two data partitions (corresponding to the two loci), the best-fit evolutionary model for each partition as determined by jMODELTEST, uncorrelated lognormal clock, a Yule process model for the species tree prior, and other parameters as default. Four independent MCMC analyses were run each for 120 million generations, sampling every 1000^th^ generation. Another MCMC analysis was run with settings suitable for a subsequent JML analysis (see below), i.e. with piecewise constant population size model and 40 million generations sampling every 1000^th^ generation. The computer program Tracer version 1.5 [53] was used to check convergence of all parameters to the stationary distribution in each run and TreeAnnotater version 1.7.4 was employed to set the burn-in (discarding the first 30000 trees) and to calculate the maximum clade credibility tree.
6. We performed statistical tree topology tests on ML gene trees inferred from the ITS_1, cpDNA and ITS_cpDNA alignments to find out whether discrepancies between topologies shown in particular gene trees and coalescent species tree are statistically significant. To this end, looking at the topology of the coalescent species tree, we enforced the following constrains on gene trees: basal position of *P. olympica* within the subsection *Hieracioides*; sister relationship of *P. nuristanica* and *P. japonica*; and monophyly of *P. hispidissima* and *P. hieracioides* (Table 1). Constrained trees were built under the evolutionary substitution model as specified for each alignment above, using the maximum likelihood (ML) criterion and heuristic search with TBR swapping algorithm and 10 random sequence addition replicates. The site-wise likelihoods for the best unconstrained ML tree and all constrained trees were calculated in the computer program raxmlGUI version 1.3 [54] and consequently were compared using the approximately unbiased, weighted Shimodaira-Hasegawa, and weighted Kishino-Hasegawa tests as implemented in the computer package CONSEL version 0.1j [55]–[57]. A *p*-value of <0.05 was chosen for rejection of the null hypothesis that the log likelihoods of the constrained and best unconstrained trees are not significantly different.
7. To test whether hybridization influenced species relationships and could be the source of gene tree incongruence, we employed the program JML [20]. This software uses a posterior distribution of species trees, population sizes and branch lengths to simulate replicate sequence datasets under the coalescent with no migration. The minimum pairwise sequence distance between sequences of two species is evaluated on the simulated datasets and compared to the one estimated from the original data (i.e. from the ITS or cpDNA dataset). This procedure, the posterior predictive test, is a good predictor of hybridization events that disturb the bifurcating species tree model. Two separate JML analyses were run to simulate sequence replicates in the ITS and cpDNA datasets. For these analyses, 40,000 species trees resulting from the JML-specified *BEAST analysis were used. Settings for particular simulations involved: (1) relative mutation rate as inferred from the log file generated during the *BEAST analyses (set to 1.016 and 0.385 for the ITS and the cpDNA simulation, respectively); (2) heredity scalar (2 and 1 for the ITS and cpDNA simulation, respectively); and (3) appropriate model of sequence evolution for both markers. In each analysis, 9,000 trees were removed as burn-in and every 10^th^ tree was used for simulations. Based on the original sequence data files, minimum pairwise sequence distances between all pairs of species and exact probabilities of observing these distances in simulations under assumption of no migration were calculated. All pairwise sequence distances with *p-*value <0.05 were recorded as potential cases of hybridization.

**Table 1:** Log likelihoods and *p*-values of AU, WSH, and WKH tests for tree comparisons considering different topological scenarios.

Topology	Alignment	Log likelihood (–ln L)	Δ (–ln L)^a^	AU^b^	WSH^c^	WKH^d^
Best maximum likelihood tree (unconstrained)	ITS_1	2408.7451	–	0.843	0.948	0.794
	cpDNA	1973.5581	–	0.777	0.903	0.650
	ITS_cpDNA	4977.4663	–	0.798	0.922	0.682
Basal position of *P. olympica* within the subsection *Hieracioides*	ITS_1	2414.1421	5.40	0.212	0.319	0.201
	cpDNA	1973.5582	0.00	0.514	0.726	0.350
	ITS_cpDNA	4979.1512	1.68	0.385	0.582	0.293
Sister relationship of *P. nuristanica* and *P. japonica*	ITS_1	2408.7453	0.00	0.250	0.709	0.206
	cpDNA	1973.5582	0.00	0.377	0.706	0.308
	ITS_cpDNA	4977.4664	0.00	0.511	0.840	0.318
Monophyly of *P. hispidissima* and *P. hieracioides*	ITS_1	2428.4956	19.75	0.013	0.077	0.057
	cpDNA	1981.1009	7.54	0.033	0.139	0.139
	ITS_cpDNA	4992.7916	15.33	0.141	0.205	0.114

Significant differences (*p*<0.05) between the best unconstrained and constrained topologies are indicated in bold.

a Δ (–ln L) - differences in log likelihoods between the best gene tree and alternative gene trees.

b AU - approximately unbiased test.

c WSH - weighted Shimodaira-Hasegawa test.

d WKH - weighted Kishino-Hasegawa test.

## Results

A total of 202 sequences of the ITS region and 205 of the cpDNA spacer of taxa a priori considered as ingroup were obtained. Results of phylogenetic analyses, however, revealed that beside of the 202 ingroup ITS sequences, another three accessions that were a priori considered as outgroup taxa (*P. scaberrima* and *P. strigosa*), appeared to be a part of ingroup. In contrast, all accessions a priori considered as outgroup taxa were shown to be part of real outgroup in the plastid region analysis (see below). Thus, sequences of 11 accessions (7 species) are considered outgroup for the ITS region while 14 accessions (9 species) for the cpDNA spacer. Information on the DNA datasets and details on maximum parsimony analyses are summarized in Table 2.

**Table 2:** Information for the DNA datasets and details on maximum parsimony analyses.

Alignment	ITS_1^a^	cpDNA^b^	concat1^c^
No. of sequences/No. of sequences in merged dataset	117	219/112	117
Characters in the alignment	653	960	1613
Length variation (including outgroup)	640–648	702–908	1352–1546
No. of variable sites	167	101	263
No. of parsimony informative characters	138	60	207
No. of most parsimonious trees	1515	1512	4130
Tree length in parsimony	252	127	430
Consistency index	0.774	0.898	0.702
Retention index	0.935	0.955	0.895

a ITS1 – analysis based on the ITS_1 Alignment S1. ITS_1.

b cpDNA - analysis based on the Alignment S3. cpDNA.

c concat - analysis based on the concatenated Alignment S4. ITS_cpDNA.

### The ITS region

In addition to homogeneous sequences, we identified numerous accessions containing intra-individual single nucleotide polymorphic sites in the ITS dataset (Table S2). Out of 653 nucleotide positions, 148 were detected to be polymorphic in at least one individual of the subsection *Hieracioides*, with up to 16 intra-individual polymorphic sites per sequence. Forty two polymorphic sites showed an additive pattern, ranging between 1 and 16 APS per individual sequence (Table S2). The heterogeneous sequences varied conspicuously in terms of number of APS with respect to potentially parental ITS variants, ranging from those with fully additive patterns to those comprising almost completely homogenized contigs.

**Figure 1:**
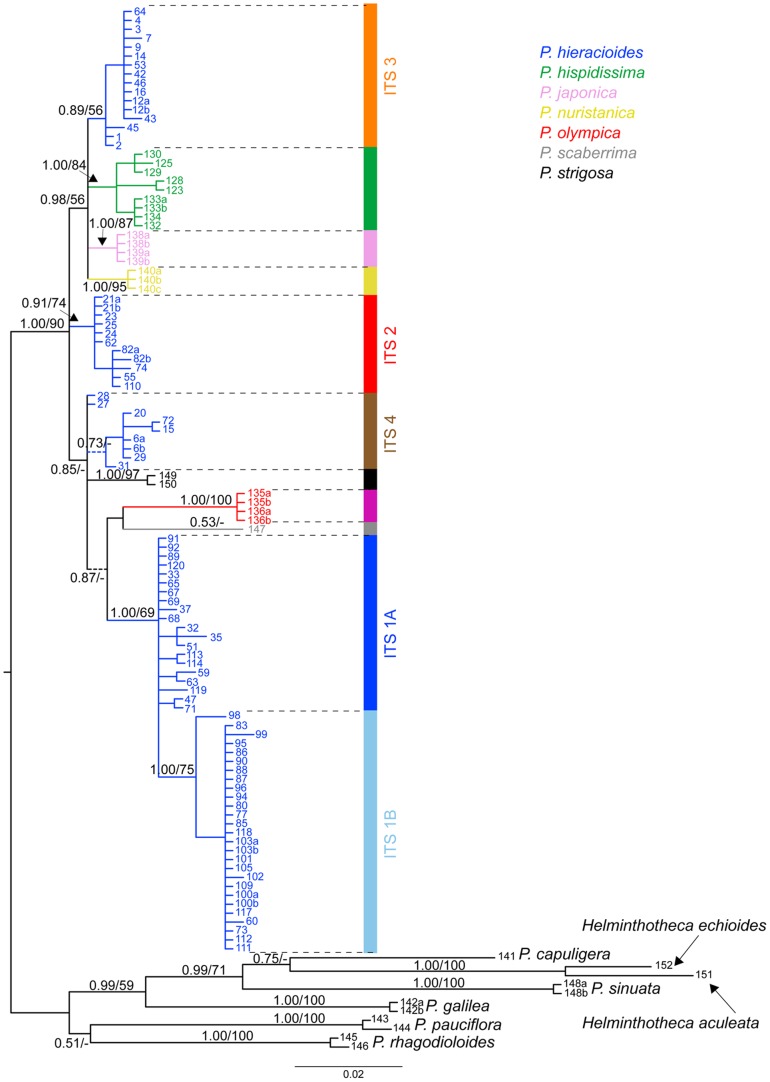
Majority-rule consensus tree of Bayesian inference based on the reduced dataset_ITS_1. The numbers above branches refer to posterior probability values of Bayesian inference/the bootstrap support as inferred for the maximum parsimony analyses (values ≥50% are shown). Support values for terminal branches are not shown. Dashed lines represent branches collapsed in strict consensus tree of the maximum parsimony analyses. Colour line below the tree indicates affiliation of grouping to the ITS genetic lineages as mentioned in the text. Each accession label includes the population code following Table S1.

**Figure 2:**
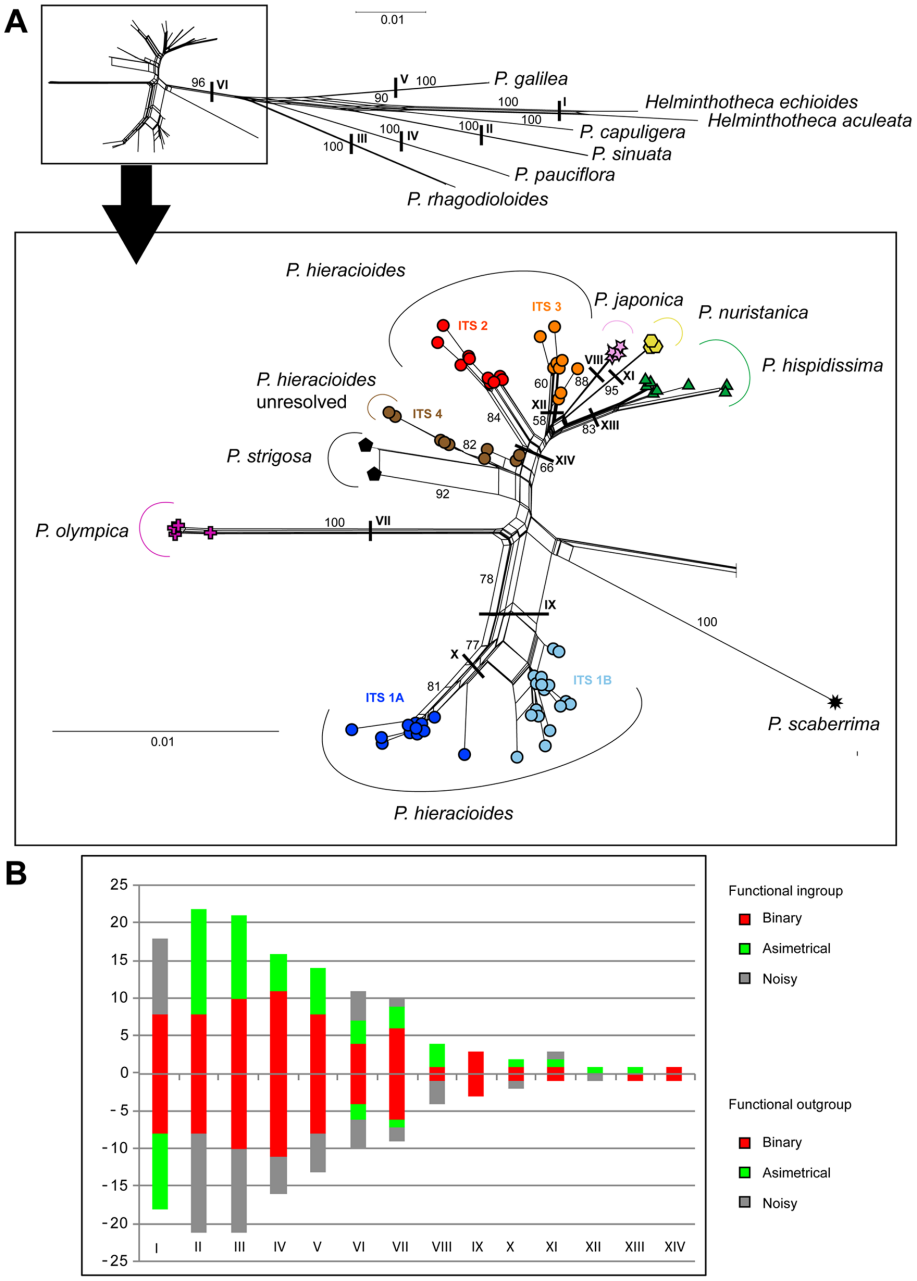
Neighbour-Net diagram (A) and split support spectrum (B) for the ITS_1 dataset. The Neighbour-Net diagram is based on uncorrected *P*-distances. Colour symbols indicate affiliation of accessions to the ITS genetic lineages as mentioned in the text while the shape of symbols indicate affiliation of accessions to taxa. Bootstrap supports of selected important splits are indicated above edges. Column height in the split spectrum represents the number of clade-supporting positions, i.e., putative primary homologies. Column parts above the y-axis represent the ingroup partition while those below the axis correspond to the outgroup partition.

Results of BI, MP and split decomposition analyses based on the ITS_1 dataset (117 sequences without APS) did not show the analysed *Picris* taxa from the subsection *Hieracioides* to form a monophyletic group (Figures 1 and 2A). Specifically, accessions of the presumed outgroup species, *P. scaberrima* and *P. strigosa*, were clustered within the ingroup taxa (Figures 1 and 2A).

**Figure 3:**
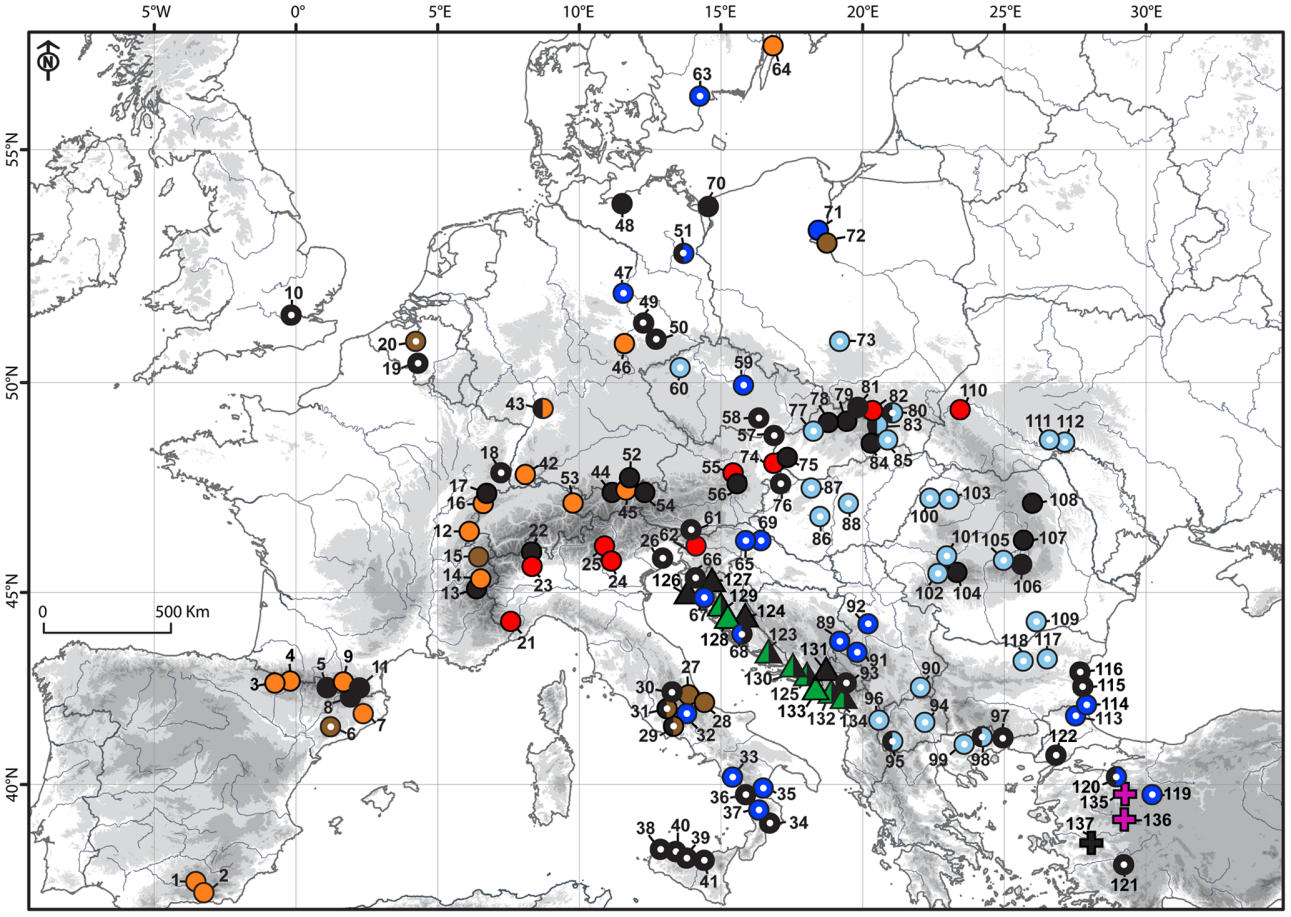
Map displaying geographic distribution of genetic variation in the analysed *Picris* taxa, as inferred from the nuclear ITS sequence data. Coloured symbols indicate affiliation of accessions to the ITS genetic lineages as mentioned in the text. Black symbols indicate populations including individuals with unhomogenised sequences containing additive polymorphic sites. Symbol shapes indicate taxa as follows: *P. hieracioides* – circle, *P. hispidissima* – triangle, and *P. olympica* – square. The circle symbols with empty centres refer to populations morphologically corresponding to *P. hieracioides* subsp. *hieracioides*, while those with solid symbols refer to populations morphologically corresponding to *P. hieracioides* subsp. *umbellata*. The populations of *P. nuristanica*, *P. japonica* and outgroup taxa are not shown. Numbers refer to population codes as denoted in Table S1.

The Bayesian 50% majority-rule consensus tree and the strict consensus MP tree based on the ITS_1 dataset (117 sequences without APS) displayed essentially identical topologies with only small differences in terminal positions and/or in clade supports (Figure 1). All ingroup taxa including both aforementioned presumed outgroup species formed a large strongly supported clade (BS = 90%, PP = 1.00), with shallow hierarchical structure comprising numerous sub-clades with various levels of support (Figure 1). All non-*hieracioides* species, *P. hispidissima*, *P. japonica*, *P. olympica*, *P. nuristanica*, *P. scaberrima*, and *P. strigosa*, formed their own strongly supported sub-clades, with *P. olympica* and *P. scaberrima* being the most divergent (Figure 1). *Picris hieracioides* is not monophyletic and forms four ITS groups with allopatric or parapatric spatial distributions (Figures 1, 2A and 3): ITS_1 composed of two sub-clades: ITS_1A – mostly lowland populations morphologically corresponding to the *P. h.* subsp. *hieracioides* morphotype from the Apennine Peninsula, the Balkan Peninsula, western Turkey, rarely from Central and northwestern Europe and a single population morphologically assignable to the *P. h.* subsp. *umbellata* morphotype from northwestern Europe (blue in Figures 1, 2A and 3); ITS_1B – mostly lowland populations of the *P. h.* subsp. *hieracioides* morphotype from Central, northwestern Europe and Balkan Peninsula and a single population of the *P. h.* subsp. *umbellata* morphotype from the Western Carpathians (pale blue in Figures 1, 2A and 3); ITS_2 – mountain populations of the *P. h.* subsp. *umbellata* morphotype from Alps and the Western Carpathians (red in Figures 1, 2A and 3); ITS_3 – mostly mountain populations corresponding to the *P. h.* subsp. *umbellata* morphotype from the Sierra Nevada, Pyrenees, Jura Mts., Alps, Germany and Sweden (orange in Figures 1, 2A and 3); and ITS_4 – an unresolved group of populations assignable to both of *P. hieracioides* morphotypes from the Apennine and the Iberian Peninsulas, Belgium and Poland (brown in Figures 1, 2A and 3). We also endeavoured to analyse the dataset ITS_2 (all 216 ITS sequences including those with APS) using MP and BI. Both approaches, however, resulted in a completely unresolved clade with all accessions from the subsection *Hieracioides* placed in a basal polytomy (data not shown).

The split decomposition diagram based on the ITS_1 dataset (117 ITS sequences) showed four main splits having various levels of bootstrap support (Figure 2A): (1) *P. olympica* separated from the rest by a set of long parallel and strongly supported edges; (2) lowland populations of *P. hieracioides*, morphologically corresponding to *P. h.* subsp. *hieracioides*, are clearly separated from all ingroup and outgroup taxa (ITS 1A and 1B). The exception represented few populations of the *P. h.* subsp. *hieracioides* morphotype that clustered together with populations attributable to the *P. h.* subsp. *umbellata* morphotype in an unresolved ITS4 group; (3) a large heterogeneous group associating several smaller well supported splits corresponding to *P. hispidissima*, *P. japonica*, *P. nuristanica*, and two lineages composed of mountainous populations of the *P. h.* subsp. *umbellata* morphotype (ITS_2 and ITS_3); and (4) a group of both morphotypes of *P. hieracioides* (ITS_4) and *P. strigosa*, a presumably outgroup species. *Picris scaberrima* formed a long edge linked with outgroup taxa by short parallel splits. Importantly, the second neighbour-net analysis based on the ITS_2 dataset (206 ITS sequences) revealed essentially the same pattern as the previous one (figure not shown). This apparently indicates that heterogeneous sequences did not significantly affect the overall split pattern. Individuals with un-homogenized ITS sequences and numerous APS appeared mostly in the central position of the network, while those possessing more homogenized sequences, with lower number of APS, were preferentially incorporated in terminal positions (figure not shown).

Analyses of spectrum of supporting positions revealed that majority of phylogenetically informative positions are shared by outgroup taxa (Figure 2B). The entire functional ingroup, involving all accessions of the subsection *Hieraciodes* plus *P. strigosa* and *P. scaberrima*, are supported only by 7 nucleotide positions. Within the functional ingroup, the highest support obtained *P. olympica* (9 positions), *P. japonica* (4 positions), the *P. hieracioides* clade with lowland populations corresponding to the *P. h. hieracioides* morphotype (3 positions) and *P. nuristanica* (2 positions). All other above mentioned splits/subclades were supported at maximum by a single position.

### The cpDNA spacer

MP and BI analyses yielded trees with different topologies to a certain extent. However, there was no strongly supported incongruence between them. In order to maintain consistency in the presentation of our results we discuss only the topology based on the BI phylogenetic tree.

**Figure 4:**
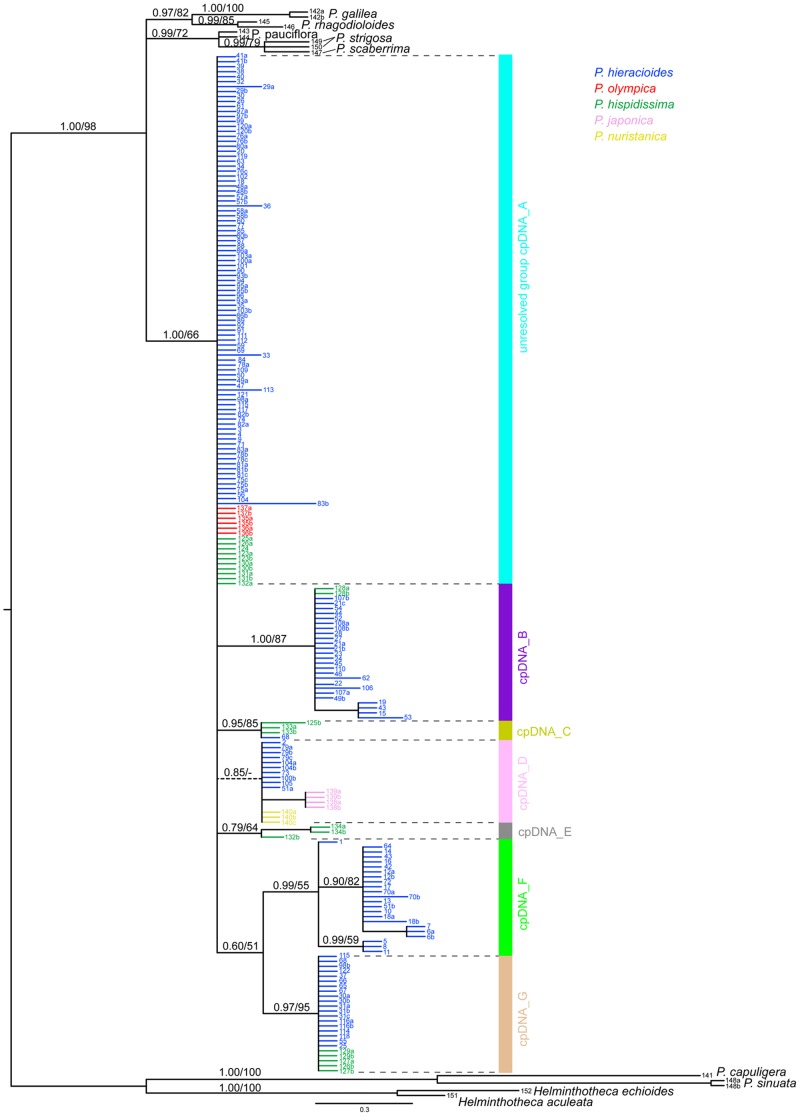
Bayesian majority-rule consensus tree based on the cpDNA intergenic spacer dataset. The numbers above branches refer to posterior probability values of Bayesian inference/the bootstrap support as inferred for the maximum parsimony analyses (values ≥50% are shown). Support values for terminal branches are not shown. Dashed lines represent branches collapsed in strict consensus tree of the maximum parsimony analyses. Colour line below the tree indicates affiliation of groupings to the cpDNA genetic lineages as mentioned in the text. Each accession label includes the population code following Table S1.

**Figure 5:**
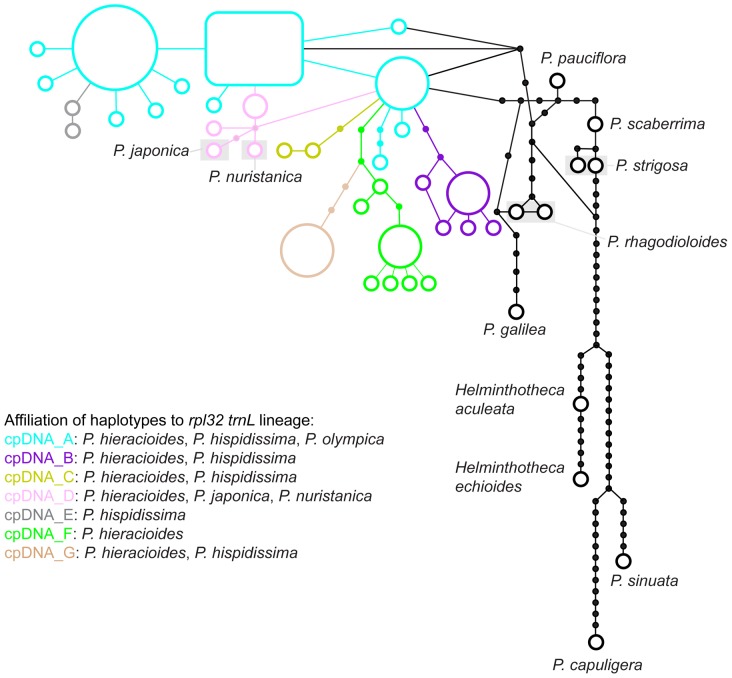
Maximum parsimony network of the cpDNA haplotypes of *Picris* populations. The symbol sizes are proportional to the haplotype frequencies, the lines represent mutational steps, and black dots are unsampled haplotypes. Colour symbols indicate affiliation of accessions to the cpDNA genetic lineages as mentioned in the text.

**Figure 6:**
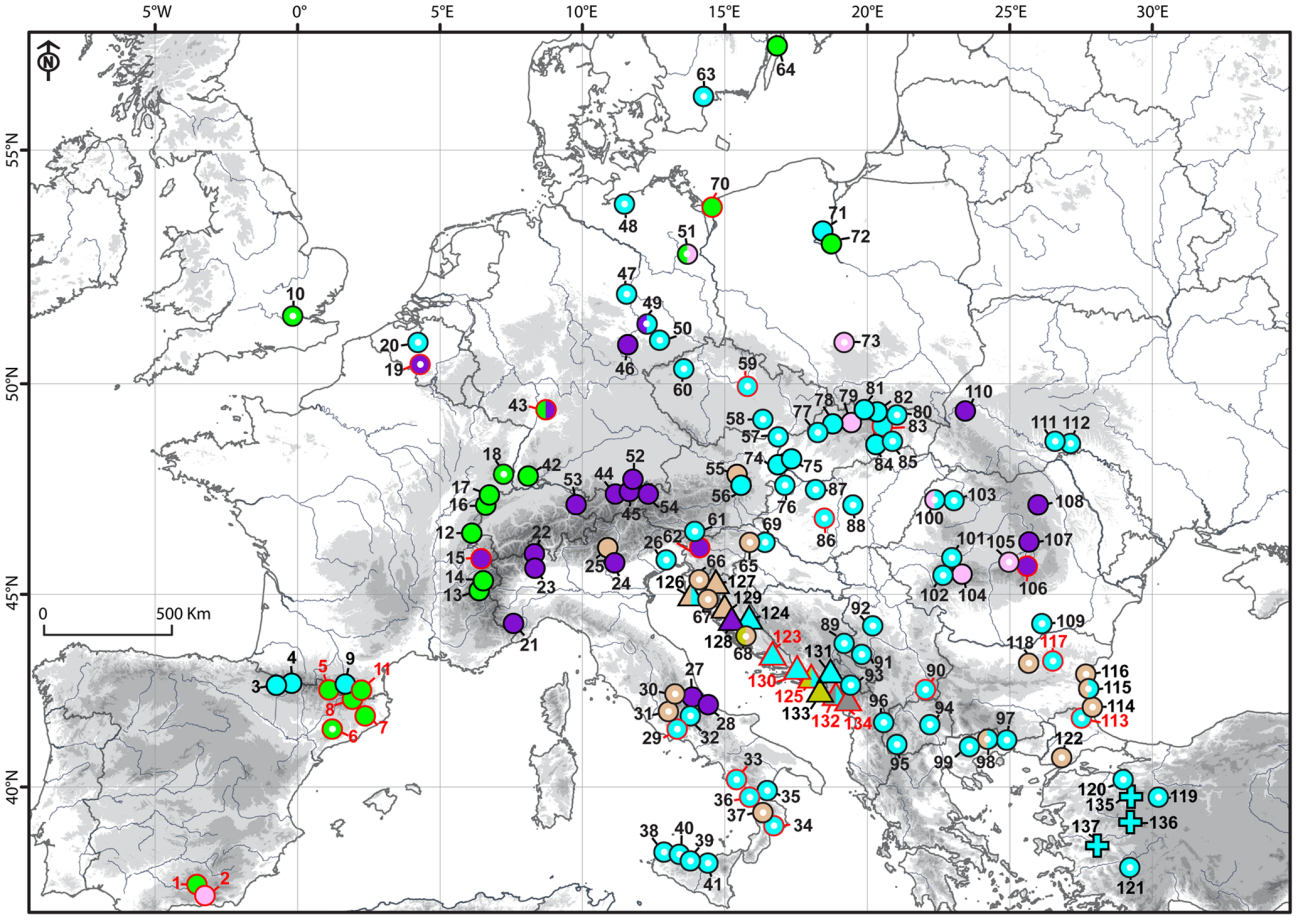
Map displaying geographic distribution of genetic variation in the analysed *Picris* taxa, as inferred from the cpDNA intergenic spacer dataset. Coloured symbols indicate affiliation of accessions to the cpDNA genetic lineages as mentioned in the text. Bi-coloured symbols denote populations possessing two different haplotypes. Symbol shapes indicate taxa as follows: *P. hieracioides* – circle, *P. hispidissima* – triangle, and *P. olympica* – square. The circle symbols with empty centres refer to populations morphologically corresponding to *P. hieracioides* subsp. *hieracioides*, while those with solid symbols refer to populations morphologically corresponding to *P. hieracioides* subsp. *umbellata.* Population of *P. nuristanica* and *P. japonica* and outgroup taxa are not shown. Numbers refer to population codes as denoted in Table S1. The symbols and their numbers highlighted in red indicate populations harbouring rare haplotypes.

In contrast to the ITS trees, *P. scaberrima* and *P. strigosa* appeared clustered together with other outgroup taxa. All accessions of the *Hieracioides* group appeared in a largely unresolved clade with five essentially supported cpDNA subclades and two unsupported groups (cpDNA_A–G) placed in a basal polytomy (Figure 4). The topology and variation of non-*hieracioides* taxa in the cpDNA spacer tree were in some aspects congruent and in other discordant with respect to the ITS tree (see Figure 1). Although not forming distinct subclades, *Picris japonica* and *P. nuristanica* possessed their own exclusive haplotypes, similarly as they did in the ITS data (see Figure 5). Contrastingly, *P. olympica* and *P. hispidissima* were heterogeneous and shared haplotypes with *P. hieracioides* (Figures 4, 5 and 6). Repeatedly, *P. hieracioides* was heterogeneous and was found in seven genetic cpDNA geographically correlated groups (Figures 4, 5 and 6): unresolved group cpDNA_A – comprising populations of *P. hieracioides* morphologically corresponding to both subspecies and originating from the entire distribution area, *P. hispidissima* from Croatia and Montenegro, and *P. olympica* (cyan in Figures 4, 5 and 6); cpDNA_B – mountain populations of the *P. h.* subsp. *umbellata* morphotype from the Apennines, Alps, and Carpathians, while populations from lowland of northwestern Europe corresponding morphologically rather to *P. h.* subsp. *hieracioides* and finally *P. hispidissima* from Croatia (violet in Figures 4, 5 and 6); cpDNA_C – *P. hispidissima* and lowland populations of the *P. h.* subsp. *hieracioides* morphotype from Croatia and Montenegro (olive in Figures 4, 5 and 6); unresolved group cpDNA_D – including mountain and lowland populations of *P. hieracioides* from Sierra Nevada, the Carpathians and north-western Europe morphologically corresponding to its both subspecies, *P. japonica* and *P. nuristanica* (pink in Figures 4, 5 and 6); cpDNA_E – *P. hispidissima* from Montenegro (grey in Figures 4, 5 and 6); cpDNA_F – populations of both morphotypes of *P*. *hieracioides* from the Iberian mountain ranges, the western Alps, the Jura Mts, and northwestern Europe (green in Figures 4, 5 and 6); cpDNA_G – lowland populations of the *P. h.* subsp. *hieracioides* morphotype from the Apennine and the Balkan Peninsula, mountain populations of the *P. h.* subsp. *umbellata* morphotype from the Alps and *P. hispidissima* from Croatia (light brown in Figures 4, 5 and 6). The highest density of rare haplotypes was observed on the Balkan Peninsula (nine haplotypes), the Iberian Peninsula mountains (seven haplotypes), and the Apennine Peninsula (five haplotypes) (Figure 6).

The parsimony-based haplotype network revealed 34 very closely related ingroup haplotypes, mostly connected by one or two steps (Figure 5) and the split decomposition diagram displayed a stellar-like structure with numerous haplotypes in an unresolved basal position (data not shown). Similarly as in the ITS dataset, the majority of supporting positions was harboured by outgroup species (data not shown). Both *hieracioides* clades as well as all their sub-clades were supported extremely weakly, in particular, by two positions at the maximum (data not shown).

### Concatenated phylogeny and coalescence based species tree

**Figure 7:**
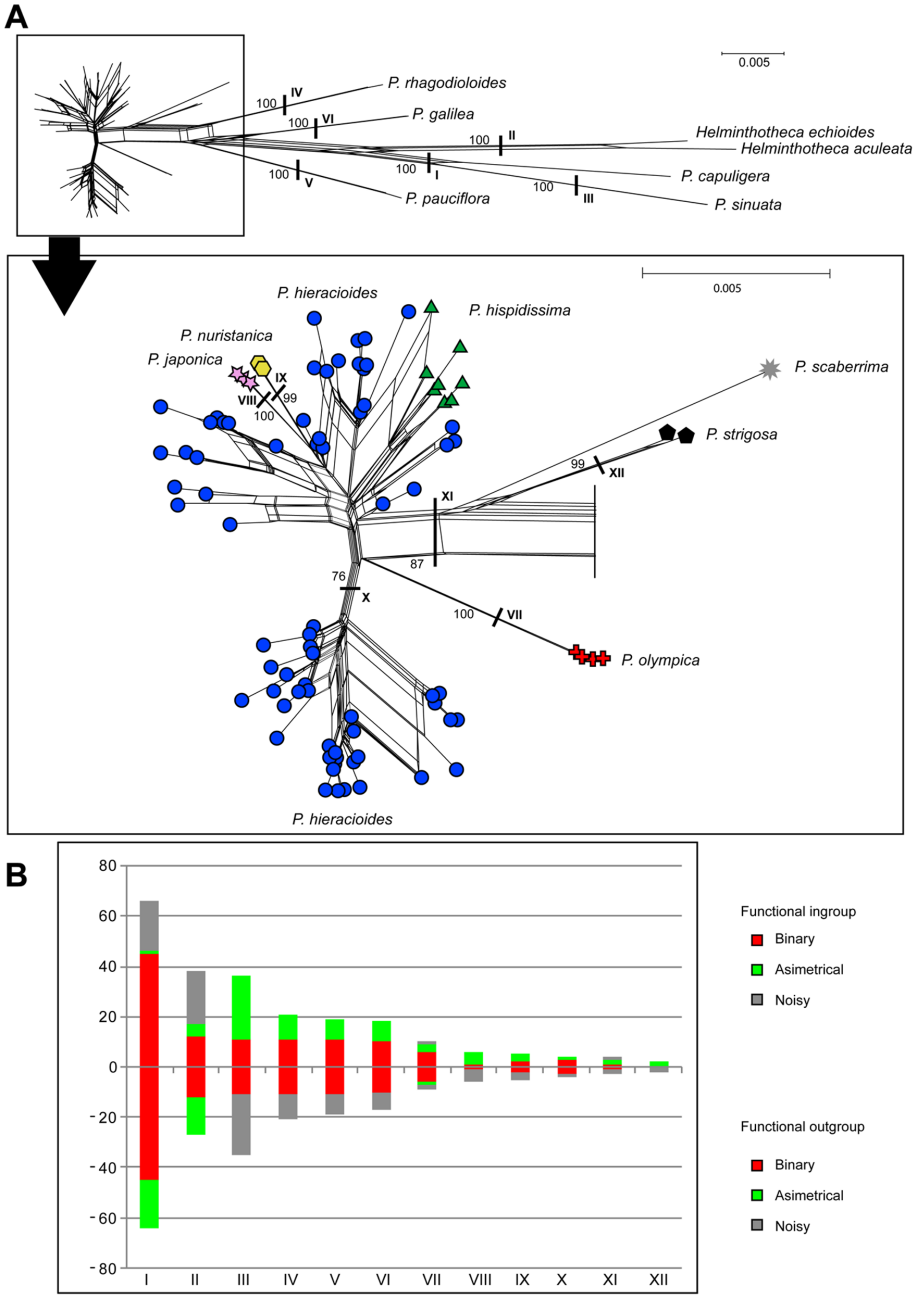
Neighbour-Net diagram (A) and split support spectrum (B) for the concatenated ITS_cpDNA dataset. Coloured symbols indicate affiliation of accessions to taxa. Bootstrap supports of selected important splits are indicated above edges. Column height in the split spectrum represents the number of clade-supporting positions, i.e., putative primary homologies. Column parts above the y-axis represent the ingroup partition while those below the axis correspond to the outgroup partition.

Although the ILD test revealed significant (*p*<0.001) incongruence between both gene trees, we performed concatenated analyses because our split spectrum analyses show that the observed discordances are not strongly supported. Thus, incongruence between both genetic regions could rather reflect paucity of phylogenetically informative signal (Figures 1, 2AB and 4). Concatenated phylogeny also did not unambiguously resolve evolutionary relationships among ingroup taxa, but still depicted several variably supported (sub)clades. Position of two presumed outgroup taxa, *P. scaberrima* and *P. strigosa*, varied among phylogenetic analyses and remained uncertain (see Figure 7AB and MP/BI phylogenies not shown). Non-*hieracioides* ingroup species, namely *P. japonica*, *P. olympica* and *P. nuristanica*, each clustered in strongly supported sub-clades, repeatedly with *P. olympica* being the most divergent (Figures 7AB). Importantly, although weakly supported, the majority of accessions assigned to morphotypes of both subspecies of *P. hieracioides* as well as to *P. hispidissima* tended to form taxon specific groupings (Figure 7A).

**Figure 8:**
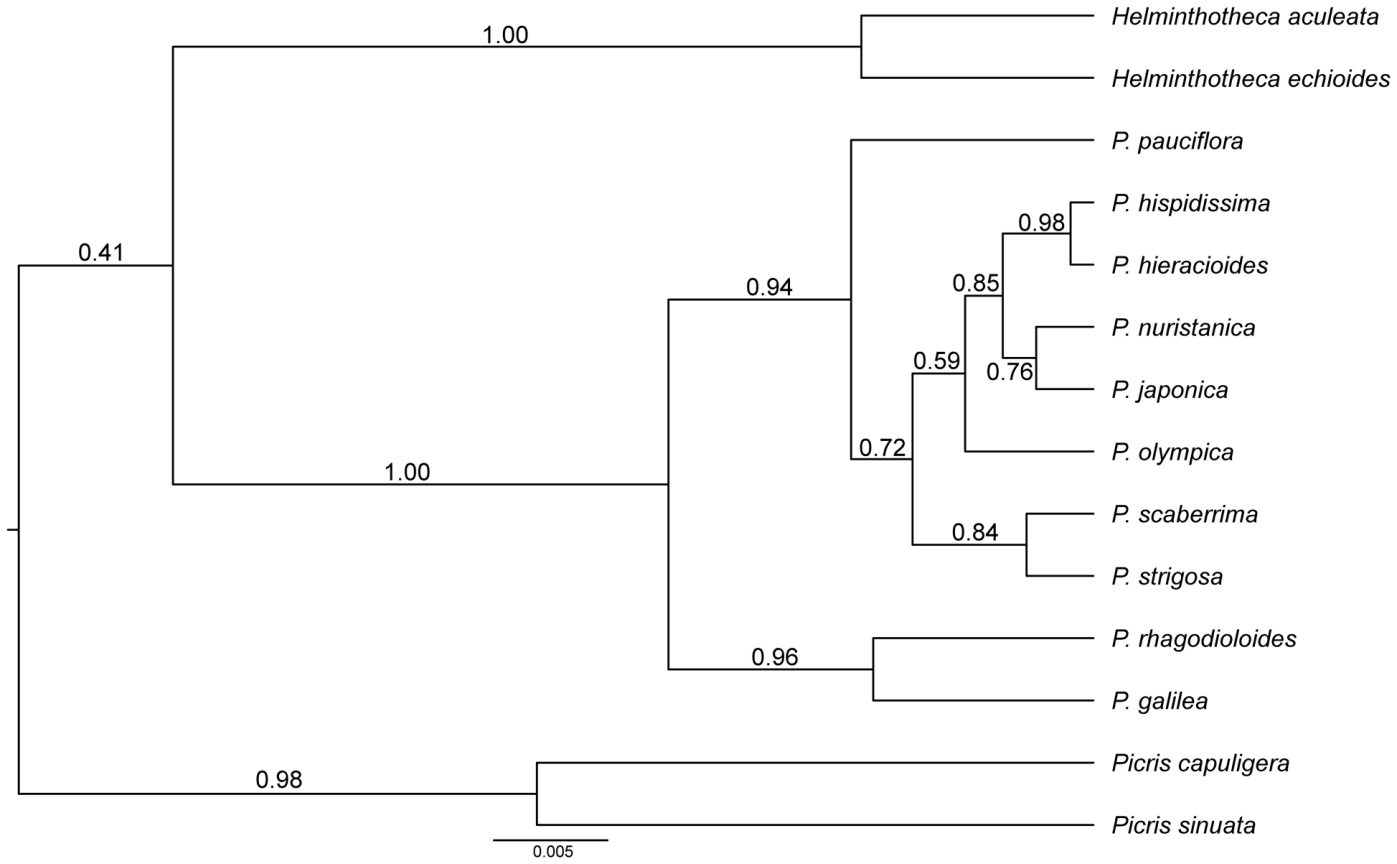
The maximum clade credibility species tree obtained from Bayesian inference in *Beast based on the ITS and cpDNA loci. The posterior probability values are indicated above the branches.

The coalescence-based species tree (Figure 8) showed different topology than the concatenated gene tree and brought more light into the phylogenetic relationships of the analysed taxa: the presumed outgroup taxa, *P. scaberrima* and *P. strigosa*, appeared together in a sister position to the subsection *Hieracioides*; *P. olympica* appeared in a basal position to all taxa from the subsection *Hieracioides*; the Asian *P. japonica* and *P. nuristanica* were clustered together in a sister position to the *P. hispidissima* and *P. hieracioides* clade. Relationships among *P. hispidissima* and *P. hieracioides* were, however, unsupported.

### Statistical tree topology tests

Although all tested evolutionary scenarios found in the coalescent species tree (see above and Material and Methods) were not observed in the present gene trees, they cannot be rejected at the significance level of 0.05 by any of the three statistical tree topology tests performed (Table 1). The only exception is the monophyly of *P. hispidissima* and *P. hieracioides*, which can be excluded for the ITS_1 and cpDNA alignment at the significance level of 0.05 only by the AU test but not by the WSH and WKH tests. Thus, the phylogenetic relationships within the subsection *Hieracioides* as unravelled by the Bayesian coalescent-based analysis cannot be very likely excluded also for the gene trees.

### Test of hybridization and/or introgression

Only a single case of potential hybridization within the ITS dataset was detected, in between a pair of outgroup taxa, *Helminthotheca aculeata* and *H. echioides* (*p*<0.001). By contrast, 31 cases with *p*<0.05 were found in the cpDNA dataset involving accessions of the corresponding pairs of the following taxa: *P. olympica* vs. *P. hieracioides*, *P. olympica* vs. *P. rhagodioloides*; *P. sinuata* vs. *P.capuligera*; and *H. aculeata* vs. *H. echioides* (Table 3).

**Table 3:** List of the distances among couples of taxa with p-values <0.05, as inferred by posterior predictive distributions.

Gene	Individual 1	Individual 2	Obs. Distance	p-value
cpDNA	*P. olympica1*	*P. h.* subsp. *hieracioides22*	0	0.0288288
	*P. olympica 1*	*P. h.* subsp. *hieracioides34*	0	0.0288288
	*P. olympica2*	*P. h.* subsp. *hieracioides22*	0	0.0288288
	*P. olympica2*	*P. h.* subsp. *hieracioides34*	0	0.0288288
	*P. olympica3*	*P. h.* subsp. *hieracioides22*	0	0.0288288
	*P. olympica3*	*P. h.* subsp. *hieracioides34*	0	0.0288288
	*P. olympica4*	*P. h.* subsp. *hieracioides22*	0	0.0288288
	*P. olympica4*	*P. h.* subsp. *hieracioides34*	0	0.0288288
	*P. olympica1*	*P. h.* subsp.*umbellata2*	0	0.0288288
	*P. olympica1*	*P. h.* subsp.*umbellata4*	0	0.0288288
	*P. olympica1*	*P. h.* subsp.*umbellata5*	0	0.0288288
	*P. olympica1*	*P. h.* subsp.*umbellata7*	0	0.0288288
	*P. olympica2*	*P. h.* subsp.*umbellata2*	0	0.0288288
	*P. olympica2*	*P. h.* subsp.*umbellata4*	0	0.0288288
	*P. olympica2*	*P. h.* subsp.*umbellata5*	0	0.0288288
	*P. olympica2*	*P. h.* subsp.*umbellata7*	0	0.0288288
	*P. olympica3*	*P. h.* subsp.*umbellata2*	0	0.0288288
	*P. olympica3*	*P. h*. subsp.*umbellata4*	0	0.0288288
	*P. olympica3*	*P. h.* subsp.*umbellata5*	0	0.0288288
	*P. olympica3*	*P. h.* subsp.*umbellata7*	0	0.0288288
	*P. olympica4*	*P. h.* subsp.*umbellata2*	0	0.0288288
	*P. olympica4*	*P. h.* subsp.*umbellata4*	0	0.0288288
	*P. olympica4*	*P. h.* subsp.*umbellata5*	0	0.0288288
	*P. olympica4*	*P. h.* subsp.*umbellata7*	0	0.0288288
	*P. rhagodioloides1*	*P. olympica1*	0.00625	0.0405405
	*P. rhagodioloides1*	*P. olympica2*	0.00625	0.0405405
	*P. rhagodioloides1*	*P. olympica3*	0.00625	0.0405405
	*P. rhagodioloides1*	*P. olympica4*	0.00625	0.0405405
	*P. sinuata1*	*P. capuligera1*	0.0197917	0.0373874
	*P. sinuata2*	*P. capuligera1*	0.0197917	0.0373874
	*H. aculeata1*	*H. echioides1*	0.00625	0.000900901
ITS	*H. aculeata1*	*H. echioides1*	0.0306279	0.00135135

## Discussion

### Intricate phylogenetic relationships within the subsect. *Hieracioides*

Tree-building and network analyses of both the nuclear and plastid markers of the studied *Picris* taxa revealed phylogenies with rather low resolutions and topological discrepancies (see Results), although utilization of these markers has been proposed as one of the most informative approaches even at lower taxonomic levels [35], [58]. More light on the evolutionary relationships within the subsection *Hieracioides* was cast by the Bayesian coalescence analysis which generates species instead of gene trees. Although relationships in the present species tree were not strongly statistically supported and were not detectable in all gene trees, they could not be rejected by statistical tree topology tests performed on the concatenated dataset (see Results). Thus, in spite of the complex patterns, we might assume the following: (1) the analysed members of the subsection *Hieracioides* have a monophyletic origin, with *P. scaberrima* and *P. strigosa* being their sister taxa; (2) *P. olympica* is basal within the subsection *Hieracioides*, i.e. it is sister to all other members of this subsection; (3) all Asian species are sister to the European ones; and (4) *P. japonica* + *P. nuristanica* are sister to *P. hieracioides* + *P. hispidissima*. Interrelationships among populations of *P. hieracioides* and *P. hispidissima* are left unresolved, however.

Which factor(s) might be responsible for low resolution, varying supports and discordances between gene tree topologies? In the present study, we have shown by the split spectrum analysis that one of the primary and crucial factors responsible for this pattern is a paucity of parsimony informative and node supporting nucleotide positions. Deficiency of phylogenetically informative signal in the datasets analysed was unambiguously evidenced by the low number of primary nucleotide homologies (Figures 2B and 7B). This was indicated also by the presence of short internal branches in all phylogenetic trees (Figures 1 and 4) and the high number of rare plastid haplotypes in derived positions connected mostly with ancestral haplotypes by only one or two mutational steps (Figure 5). Low level of phylogenetically informative positions in the used markers, which are among the most informative [35], [58], strongly indicates a recent diversification of the studied taxa and genetic groups [1], [59], [60], [61], [62]. It would be desirable to confirm this by molecular dating. However, in case of insufficient amount of phylogenetic information, such an analysis is assumed to be vague and statistically unsupported.

Furthermore, incomplete lineage sorting represents another evolutionary force that might be responsible for the discordances and complex genetic patterns in the six *Picris* taxa [1], [3], [4], [5]. The Bayesian coalescent-based approach considers the incomplete lineage sorting as a main source of uncertainties in inferred species trees [18]. Thus, the unresolved relationships among genetic lineages of *P. hieracioides* and *P. hispidissima* as well as the low nodal support could signalise either influence of this phenomenon or violation of prior assumption by hybridisation or other processes. Likewise, the disjunctive occurrence of the ancestral plastid haplotypes in populations of *P. olympica* and the spatially distant *P. hieracioides* might be the consequence of incomplete lineage sorting. By contrast, the mosaic pattern detected in the ITS dataset may repeatedly suggest influence of hybridization.

Hybridisation might substantially influence phylogenetic reconstructions at even lower hierarchical levels. Decreased phylogenetic tree resolution, incongruent patterns in different gene trees, and occurrence of alleles transcending taxa or genetic groups are commonly observed in plant phylogenies in which extensive gene exchange between taxa has been documented [2], [3], [17], [41], [60], [61], [62], [63], [64], [65], [66]. Presence of extensive and recurrent gene exchange among investigated *Picris* taxa might be indicated by the following facts. (1) Considerable intra-individual and intra-population ITS sequence heterogeneity and allele transcending clades and taxon borders in *P. hieracioides*, *P. hispidissima*, and *P. olympica*. On the other hand, maintenance of two or more ITS variants within a single genome might be attributed to other molecular-genetic processes [11], [67]. (2) Occurrences of heterogeneous ITS sequences predominantly in the contact zones of the studied species or genetic groups, while locations of homogeneous sequences with little or no genetic admixture mostly along the sampled area's margins inhabited mainly by single lineages or species (see Results, Figure 3). (3) Strict self-incompatibility in *P. hieracioides* and *P. hispidissima*, which has been recently documented ([27]; M. Slovák unpublished data). (4) Last but not least, successful crossing between populations of morphotypes and genetic lineages of *P. hieracioides* as well as with *P. hispidissima* obtained during our preliminary field experiments (M. Slovák, unpublished data).

Posterior predictive checking revealed hybridization/introgression at least in five pairs of the studied species. Four pairs were recorded exclusively by simulations on the cpDNA dataset and one pair was detected by simulations on both the plastid and the nuclear dataset. Since the method of posterior predictive checking is powerful unless the hybridization event has occurred very rapidly after the speciation event [9], it might be hypothesised that hybridizations among the *Picris* taxa are rather recent or have occurred later after speciation events. This hypothesis corresponds well mostly in the case of comparatively distantly related species, *P. olympica* with *P. hieracioides*, *P. olympica* with *P. rhagodioloides*, and *P. capuligera* with *P. sinuata*. Such hybridisation events might take part during the Pleistocene glacial/interglacial range shifts, especially, at sites where lowland taxa could have had recurrent contact with mountainous ones [2], [68]. Posterior predictive checking, however, did not support hybridisation scenarios among the pairs of closest relative taxa where it would be expected: among subspecies and genetic lineages of *P. hieracioides* as well as between *P. hieracioides* and *P. hispidissima*. One of the most plausible explanations might be that the hybridization events among those taxa happened just after their diversification. Especially in case of the two subspecies of *P. hieracioides*, the divergence of genetic lineages might be shallow causing hybridization to be undetectable [9].

The overall failure to detect hybridization events by simulations on the ITS dataset might be attributed to the presence of recombination and/or concerted evolution within the ITS locus. As stated in [9], some patterns resulting from concerted evolution can potentially bias the estimates of population sizes toward lower values. This causes the loci to coalesce faster and therefore hybridization might be harder to detect. Only one hybridization event was found by both genetic markers, viz., in *H. aculeata* and *H. echioides*. The sample size might be the limitation factor in the posterior predictive checking approach. Since only a single individual per each aforementioned species (outgroup) was sampled, their population sizes cannot be properly estimated for species tree reconstruction. Therefore we assume this result might be a methodological artefact.

Finally, it remains arguable whether some of the studied taxa, but especially *P. hispidissima*, fit into the reticulate evolution scenario or, alternatively, its large genetic heterogeneity arose from genetic erosion. The latter hypothesis might be explained by recurrent introgression with adjacent populations of *P. hieracioides* after their diversification. We are more inclined to the latter scenario because all taxa are morphologically rather well delimited, inhabit ecologically specific biotopes, and trans-taxon alleles are concentrated predominantly in areas of sympatry or parapatry.

Although methods to potentially distinguish between hybridisation and incomplete lineage sorting have been developed, there is still a high risk of confusing these two phenomena or they cannot be discerned in some cases at all [9], [12], [13]. In the investigated closely related *Picris* taxa from the subsection *Heiracioides*, it is highly tricky or even impossible to discern between these two processes. Reasons for this ambiguity might come from weak genetic divergence of analysed taxa, and importantly from presence of gene transfer via introgression which, moreover, most probably occurred repeatedly during their evolutionary history.

### Comparison of genetic patterns with results inferred from AFLP and morphology

Spatial distribution of genetic variation in populations of *P. hieracioides* and *P. hispidissima* varied considerably across phylogenetic trees, with both concordant and discordant patterns being detected (Figures 1 and 4). The ITS data were highly consistent with taxonomic/morphological delimitation of the studied taxa. The genetic ITS groups of all studied species as well as within *P. hieracioides* exhibited high levels of concordance with the results of the AFLP analyses [24]. The congruence of genetic patterns resolved by ITS and AFLP, especially, with regard to the phylogenetic signal, has been already demonstrated for various plant groups [69]. The spatial distribution of genetic variation in populations and genetic lineages of *P. hieracioides* and in *P. hispidissima* was, however, clearly blurred by populations harbouring individuals with non-homogenized ITS sequences. Such a diffuse pattern of genetic variation disrupting geographic structure is not unexpected in the nuclear biparentally inherited genetic markers. In fact, this reflects both seed and pollen-mediated gene dispersal [70], especially, in widespread taxa with unrestricted gene flow [71]–[73]. Numerous heterogeneous ITS sequences did not, however, display APS for some positions variable in the potentially parental haplotypes (Table S2). This pattern can be attributed to in vivo recombination between ITS ribotypes, or to the eventual combination of minor, undetected alleles with more widespread ones [11], [67], [74]. Detection of such minor ITS ribotypes within taxa/populations might be prevented by preferential amplification of the major ITS copies or by quantitative masking of the rare ribotypes [74]. Another highly plausible explanation on the origin of incomplete homogenization of APS involves concerted evolution [2], [40], [58], [63], [64], [67]. Its presence in our ITS dataset might be one of the crucial reasons responsible for the failure to detect mutual hybridisation among populations and genetic lineages of *P. hieracioides* and *P. hispidissima* (see discussion above).

In contrast, genetic variation inferred from the plastid cpDNA spacer was, however, only partly congruent with that captured by ITS, AFLPs, and morphology. Further, numerous discrepancies in the number and composition of the genetic cpDNA groups were noted (see ‘Results’). Thus, the plastid pattern exhibited an apparently mosaic-like spatial structure and two or more species/genetic groups shared same plastid haplotypes. This was, especially, true for the sympatric/parapatric populations (Figure 6). Complex and incongruent genetic plastid patterns might, beside of the aforementioned hybridisation and incomplete lineage sorting, arise from different attributes of the employed genetic markers such as their evolutionary rate, inheritance mode (bi-parental vs. uni-parental), or number of copies (multiple vs. single copies), which reflects different time horizons of evolution [11]. The nuclear ITS marker evolves faster than plastid ones and therefore enable detection of more recent diversification events [11], [75].

### Evolution of the studied *Picris* taxa and taxonomic implications

Our results suggested that evolution of the studied *Picris* species was shaped by the interplay of several factors and evolutionary processes. Their diversification was most likely rapid, and may have occurred in an allopatric manner. Since all of these taxa, but especially, *P. hispidissima* and *P. olympica*, have unique habitat and ecology preferences (see Material and Methods), we presume that ecological factors also played an important role in their speciation.

The centre of the diversity of the studied species complex, as indicated by the distributions of *P. olympica* and *P. hispidissima* as well as by the accumulation of rare haplotypes, lies in the southern European peninsulas and partly also the Alps and the Carpathians (Figure 6). These regions have been shown to be the most important glacial refugia for the majority of European flora and fauna [76], [77]. This might suggest that the evolutionary history of the investigated *Picris* taxa was connected with glacial/interglacial cycles in the Quaternary. This is in concordance with conclusions concerning the origin of the AFLP lineages, as detected within *P. hieracioides* [24].

Genetic data presented herein together with recent distribution [21] indicate that *P. olympica* very likely evolved in alpine levels of high mountain ranges of Anatolia in Asia Minor. Here it very likely survived isolated for a long period, although remote secondary contacts and interaction with other closely related taxa cannot be excluded. Likewise, autecological characteristics and distribution of *P. hispidissima* suggest that evolutionary history of this taxon has undoubtedly been confined with coastal, calcareous mountain ranges of the western part of the Balkan Peninsula. This region has also been repeatedly proven as important evolutionary hotspot and glacial refugium for other plant groups [32], [68].

Furthermore, genetic lineages detected within *P. hieracioides* most likely diversified in allopatry or parapatry in southern European peninsulas and adjacent mountainous regions of the Alps and the Carpathians. The mountain-dwelling populations morphologically corresponding to the *P. h.* subsp. *umbellata* most likely evolved in European mountains or their proximity (the Alps, central Apennines, the Carpathians, Iberian mountain ranges and the Jura Mts.) [24]. If it had existed there already during Pleistocene climatic oscillations, it could potentially survive in the peripheral glacial areas together with other mesophylous herbs [78], [79]. As proved by the highest cpDNA haplotype richness, diversification centre of populations morphologically assignable to the lowland *P. h.* subsp. *hieracioides* was located more southwards, especially, in lowland and hilly regions of the Apennine Peninsula and plausibly also in the Balkan Peninsula. Subsequently, they colonised other habitats of their present day occurrence in central and north-western Europe as well as in Asia Minor from these southern refugia.

An important taxonomic question is whether the analysed taxa should be recognised as distinct species/subspecies, or whether they rather represent informal phylogeographic lineages within a single heterogeneous taxon. The latter possibility has been favoured in case of several other complex plant species groups [30], [32], [80], [81]. *Picris hieracioides* has been here evidenced to be highly heterogeneous across both markers. Actually, both plastid and ITS nuclear alleles have transcended borders of both subspecies of *P. hieracioides*, as recently delimited in [24]. Moreover, several populations of *P. hieracioides* displayed transitional morphological variation. In addition, from ecological point of view, *P. hieracioides* is characterised by an exceptionally large range of habitat preferences [22], [24]. All these facts might indicate that both currently delimited subspecies of *P. hieracioides* are artificial biological units, representing rather ecotypes evolved recurrently in different evolutionary lineages under similar environmental influences than real taxa.

Ecotype hypothesis, however, strongly contradicts results of our previous study focused not only on investigation of wild populations of *P. hieracioides*, but also on cultivation experiments realised under environmentally homogeneous conditions [22], [23], [24]. These experiments included more than 300 individuals from 32 populations that belonged to both subspecies of *P. hieracioides*, originating from almost the entire studied area [24]. Subsequent comparative morphological investigations revealed that both subspecies retained morphologically stable under homogeneous environmental conditions. Importantly, these analyses showed that environmental conditions have had only a minute impact on the morphological characters identified as taxonomically diagnostic. Likewise, *P. hispidissima* retained its morphological distinctiveness under environmentally homogenous conditions (M. Slovák, unpublished data). Thus, taxonomically important morphological features of these entities are not a consequence of environmental plasticity, but are genetically inherited instead. All three taxa are well defined morphologically, ecologically and partially also genetically (AFLP's) at least within ‘pure’ populations untouched by their recent mutual secondary contacts.

Both, subspecies of *P. hieracioides* as well as *P. hispidissima* are recently diversified and thus genetically weakly separated taxa. Additionally, their genetic variation has been significantly affected by their reciprocal multiple secondary contacts, taking place after anthropogenically mediated range expansions. Due to the lack of habitat and breeding isolation barriers, hybridisation and introgression among these taxa occur frequently in contact zones. This process leads to the genetic and morphological homogenisation of affected populations, erasing their distinctness in contact zones. Similar cases of taxon fusion caused by hybridisation preceded by migration of populations enhanced by human activities have been reported for instance in members of the genus *Knautia* [80] and *Cerasus* [82]. For the latter case the term “anthropohybridisation” was coined. Establishment of sound taxonomic concept in such difficult cases is very problematic and usually it is a matter of subjective choice. Therefore, at the present state of knowledge, we prefer to leave both subspecies of *P. hieracioides* at currently accepted taxonomic ranks [24]. In contrast, *P. hispidissima* should be included under highly variable *P. hieracioides* as separated subspecies: *P. h.* subsp. *hispidissima* (Bartl.) Slovák and Kučera, comb. nova, hoc loco (basionym: *Crepis hispidissima* Bartl. in Bartling & Wendland, Beitr. Bot. 2: 125, 1825).

Although this study does not provide comprehensive and firm taxonomic conclusions, it represents an important background for future investigations on this intricate and dynamically evolving species complex. Last but not least, the present investigation documents rapid process of taxon erosion, happening due to the anthropogenic disturbance of natural biotopes.

## Supporting Information

Table S1 **Locality details of studied taxa, number of individuals used for the particular molecular analyses, and GenBank accession numbers.**
(DOC)

Table S2 **Summary of nucleotide site variation for the nuclear ITS region in the studied taxa.**
(XLS)

Alignment S1 **ITS_1: comprised 117 ITS sequences without APS.** The accession labels in alignments follow the population and taxon codes used in Table S1.
(TXT)

Alignment S2 **ITS_2: comprised all 206 ITS sequences.** The accession labels in alignments follow the population and taxon codes used in Table S1.
(TXT)

Alignment S3 **cpDNA: comprised all 219 rpl32-** ***trn*** **L^UAG^ sequences.** The accession labels in alignments follow the population and taxon codes used in Table S1.
(TXT)

Alignment S4 **ITS_cpDNA: comprised 117 ITS sequences without APS and corresponding plastid ones.** The accession labels in alignments follow the population and taxon codes used in Table S1.
(TXT)

## References

[1] Comes HP, Abbott RJ (2001) Molecular phylogeny, reticulation, and lineage sorting in Mediterranean *Senecio* sect. *Senecio* (Asteraceae). Evolution 55: 1943–1962. 11761056

[2] Nieto Feliner G, Gutiérrez Larena B, Fuertes Aguilar J (2004) Fine-scale geographical structure, intra-individual polymorphism and recombination in nuclear ribosomal internal transcribed spacers in *Armeria* (Plumbaginaceae). Ann Bot (Oxford) 93: 189–200. 10.1093/aob/mch027 PMC4241081 14707002

[3] Willyard A, Cronn R, Liston A (2009) Reticulate evolution and incomplete lineage sorting among the ponderosa pines. Molec Phylogen Evol 52: 498–511. 10.1016/j.ympev.2009.02.011 19249377

[4] Schmidt-Lebuhn AN, de Vos JM, Keller B, Conti E (2012) Phylogenetic analysis of *Primula* section *Primula* reveals rampant non-monophyly among morphologically distinct species. Molec Phylogen Evol 65: 23–34. 10.1016/j.ympev.2012.05.015 22643289

[5] Yu WB, Huang PH, Li DZ, Wang H (2013) Incongruence between nuclear and chloroplast DNA phylogenies in *Pedicularis* section *Cyathophora* (Orobanchaceae). PLoS ONE, doi: 10.1371/journal.pone.0074828 10.1371/journal.pone.0074828 PMC3777957 24069353

[6] Rieseberg LH (1997) Hybrid origins of plant species. Ann Rev Ecol Syst 28: 359–389.

[7] Wendel JF, Doyle J (1998) Phylogenetic incongruence: Window into genome history and molecular evolution. In: Soltis D, Soltis P, Doyle J, editors.Molecular systematics of plants II: DNA sequencing. Kluwer, Boston, USA. pp. 265–296.

[8] Linder CR, Rieseberg LH (2004) Reconstructing patterns of reticulate evolution in plants. Amer J Bot 91: 1700–1708. PMC2493047 18677414

[9] Joly S, McLenachan PA, Lockhart PJ (2009) A statistical approach for distinguishing hybridisation and incomplete lineage sorting. Amer Nat 174: 54–70. 10.1086/600082 19519219

[10] Funk DJ, Omland KE (2003) Species-level paraphyly and polyphyly: Frequency, causes, and consequences, with insights from animal mitochondrial DNA. Annual Rev Ecol Evol Syst 34: 397–423.

[11] Small RL, Cronn RC, Wendel JF (2004) Use of nuclear genes for phylogeny reconstruction in plants. Austral Syst Bot 17: 145–170.

[12] Holland BR, Benthin S, Lockhart PJ, Moulton V, Huber KT (2008) Using supernetworks to distinguish hybridisation from lineage-sorting. BMC Evol Biol 8: 202. 18625077 10.1186/1471-2148-8-202 PMC2500029

[13] Yu Y, Than C, Degnan JH, Nakhleh L (2011) Coalescent histories on phylogenetic networks and detection of hybridisation despite incomplete lineage sorting. Syst Biol 60: 138–149. 21248369 10.1093/sysbio/syq084 PMC3167682

[14] Wiens JJ, Hollingsworth BD (2000) War of the iguanas: Conflicting molecular and morphological phylogenies and long-branch attraction in iguanid lizards. Syst Biol 49: 143–159. 12116477 10.1080/10635150050207447

[15] Duvall MR, Ervin AB (2004) 18S gene trees are positively misleading for monocot/dicot phylogenetics. Molec Phylogen Evol 30: 97–106. 10.1016/s1055-7903(03)00187-8 15022761

[16] van der Niet T, Linder HP (2008) Dealing with incongruence in the quest for the species tree: a case study from the orchid genus *Satyrium* . Molec Phyl Evol 47: 154–174. 10.1016/j.ympev.2007.12.008 18325794

[17] Frajman B, Eggens F, Oxelman B (2009) Hybrid origins and homoploid reticulate evolution within *Heliosperma* (Sileneae, Caryophyllaceae) - A multigene phylogenetic approach with relative dating. Syst Biol 58: 328–345. 20525587 10.1093/sysbio/syp030

[18] Heled J, Drummond AJ (2010) Bayesian inference of species trees from multilocus data. Molec Biol Evol 27: 570–580. 19906793 10.1093/molbev/msp274 PMC2822290

[19] Kubatko L, Carstens B, Knowles L (2009) STEM: species tree estimation using maximum likelihood for gene trees under coalescence. Bioinformatics 25: 971–973. 19211573 10.1093/bioinformatics/btp079

[20] Joly S (2012) JML: testing hybridization from species trees. Molec Ecol Res 12: 179–184. 10.1111/j.1755-0998.2011.03065.x 21899723

[21] Lack HW (1974)Die Gattung *Picris* L. sensu lato im ostmediterran–westasiatischen Raum. PhD thesis, University of Vienna, Austria. 184 p.

[22] Slovák M, Marhold K (2007) Morphological evaluation of *Picris hieracioides* L. (Compositae) in Slovakia. Phyton (Horn) 47: 73–102.

[23] Slovák M, Urfus T, Vít P, Marhold K (2009) Balkan endemic *Picris hispidissima* (Compositae): Morphology, DNA content and relationship to polymorphic *P. hieracioides* . Pl Syst Evol 278: 178–201.

[24] Slovák M, Kučera J, Marhold K, Zozomová-Lihová J (2012) The morphological and genetic variation in the polymorphic species *Picris hieracioides* (Compositae, Lactuceae) in Europe strongly contrasts with traditional taxonomical concepts. Syst Bot 21: 258–278.

[25] Lack HW (1979) The genus *Picris* (Asteraceae, Lactuceae) in Tropical Africa. Pl Syst Evol 131: 35–52.

[26] Samuel R, Gutermann W, Stuessy TF, Ruas CF, Lack HW, et al (2006) Molecular phylogenetics reveals *Leontodon* (Asteraceae, Lactuceae) to be diphyletic. Amer J Bot 93: 1193–1205. 21642184 10.3732/ajb.93.8.1193

[27] Slovák M, Šingliarová B, Mráz P (2008) Chromosome numbers and mode of reproduction in *Picris hieracioides* s.l. (Asteraceae) with notes on some other *Picris* taxa. Nordic J Bot 28: 238–244.

[28] Slovák M, Vít P, Urfus T, Suda J (2009) Complex pattern of genome size variation in a polymorphic member of the Asteraceae. J Biogeogr 36: 372–384.

[29] Lack HW (1975) *Picris*. In: Davis PH, editor.Flora of Turkey and the East Aegean Islands 5.Edinburgh, UK: University Press. pp. 678–684.

[30] Frajman B, Oxelman B (2007) Reticulate phylogenetics and phytogeographical structure of *Heliosperma* (Sileneae, Caryophyllaceae) inferred from chloroplast and nuclear DNA sequences. Molec Phylogen Evol 43: 140–155. 10.1016/j.ympev.2006.11.003 17188521

[31] Prentice HC, Malm JU, Hathaway L (2008) Chloroplast DNA variation in the European herb *Silene dioica* (red campion): postglacial migration and interspecific introgression. Pl Syst Evol 272: 23–37.

[32] Bardy KE, Albach DC, Schneeweiss GM, Fischer MA, Schönswetter P (2010) Disentangling phylogeography, polyploid evolution and taxonomy of a woodland herb (*Veronica chamaedrys* group, Plantaginaceae s.l.) in southeastern Europe. Molec Phylogen Evol 57: 771–786. 10.1016/j.ympev.2010.06.025 PMC2989448 20603220

[33] Shaw J, Lickey EB, Schilling EE, Small RL (2007) Comparison of whole chloroplast genome sequences to choose noncoding regions for phylogenetic studies in angiosperms: The tortoise and the hare III. Amer J Bot 94: 275–288. 21636401 10.3732/ajb.94.3.275

[34] Chapman MA, Chang JC, Weisman D, Kesseli RV, Burke JM (2007) Universal markers for comparative mapping and phylogenetic analysis in the Asteraceae (Compositae). Theor Appl Genet 115: 747–755. 17634914 10.1007/s00122-007-0605-2

[35] Shaw J, Lickey EB, Beck JT, Farmer SB, Liu W, et al (2005) The tortoise and the hare II: Relative utility of 21 noncoding chloroplast DNA sequences for phylogenetic analysis. Amer J Bot 92: 142–166. 21652394 10.3732/ajb.92.1.142

[36] Francisco-Ortega J, Fuertes Aguilar J, Gómez Campo C, Santos-Guerra A, Jansen RK (1999) Internal transcribed spacer sequence phylogeny of *Crambe* L. (Brassicaceae): Molecular data reveal two old world disjunctions. Molec Phylogen Evol 11: 361–380. 10.1006/mpev.1998.0592 10196078

[37] White TJ, Bruns T, Lee S, Taylor JW (1990) Amplification and direct sequencing of fungal ribosomal RNA genes for phylogenetics. In: Innis MA, Gelfand DH, Sninsky JJ, White TJ, editors.PCR Protocols: A Guide to Methods and Applications.New York, USA: Academic Press. pp. 315–322.

[38] Timme R, Kuehl EJ, Boore JK, Jansen RK (2007) A comparative analysis of the *Lactuca* and *Helianthus* (Asteraceae) plastid genomes: identification of divergent regions and categorization of shared repeats. Amer J Bot 94: 302–313. 21636403 10.3732/ajb.94.3.302

[39] Hall TA (1999) BioEdit: a user-friendly biological sequence alignment editor and analysis program for Windows 95/98/NT. Nucleic Acids Symp Ser (Oxford) 41: 95–98.

[40] Fuertes Aguilar J, Nieto Feliner G (2003) Additive polymorphisms and reticulation in an ITS phylogeny of thrifts (*Armeria*, Plumbaginaceae). Molec Phylogen Evol 28: 430–447. 10.1016/s1055-7903(02)00301-9 12927129

[41] Záveská E, Fér T, Šída O, Krak K, Marhold K, et al (2012) Phylogeny of *Curcuma* (Zingiberaceae) based on plastid and nuclear sequences: Proposal of the new subgenus *Ecomata* . Taxon 61: 747–763.

[42] Farris JS, Källersjö M, Kluge AG, Bult C (1994) Testing significance of congruence. Cladistics 10: 315–319.

[43] Swofford DL (2001) PAUP*. Phylogenetic Analysis Using Parsimony (*and Other Methods), v. 4.0 beta 10. Sunderland: Sinauer Associates.

[44] Maddison DR, Maddison WP (2000)MacClade4: analysis of phylogeny and character evolution, version 4.0. Sinauer, Sunderland, Massachusetts, USA. 398 p.

[45] Ronquist F, Huelsenbeck JP (2003) MrBayes 3: Bayesian phylogenetic inference under mixed models. Bioinformatics 19: 1572–1574. 12912839 10.1093/bioinformatics/btg180

[46] Miller MA, Pfeiffer W, Schwartz T (2010) “Creating the CIPRES Science Gateway for inference of large phylogenetic trees” in Proceedings of the Gateway Computing Environments Workshop (GCE), 14 Nov. 2010, New Orleans, LA : 1–8 pp.

[47] Guindon S, Gascuel O (2003) A simple, fast, and accurate algorithm to estimate large phylogenies by maximum likelihood. Syst Biol 52: 696–704. 14530136 10.1080/10635150390235520

[48] Posada D (2008) ModelTest: Phylogenetic Model Averaging. Molec Biol Evol 25: 1253–1256. 18397919 10.1093/molbev/msn083

[49] Huson DH, Bryant D (2006) Application of phylogenetic networks in evolutionary studies. Molec Biol Evol 23: 254–267. 16221896 10.1093/molbev/msj030

[50] Templeton AR, Crandall KA, Sing CF (1992) A cladistic analysis of the phenotypic associations with haplotypes inferred from restriction endonuclease mapping and DNA sequence data. III. Cladogram estimation. Genetics 132: 619–633. 1385266 10.1093/genetics/132.2.619 PMC1205162

[51] Clement M, Posada D, Crandall K (2000) TCS: a computer program to estimate gene genealogies. Mol Ecol 9: 1657–1660. 11050560 10.1046/j.1365-294x.2000.01020.x

[52] Wägelle JW, Rödding F (1998) A priori estimation of phylogenetic information conserved in aligned sequences. Molec Phylogen Evol 9: 358–365. 10.1006/mpev.1998.0501 9667983

[53] Rambaut A, Drummond AJ (2007) Tracer v1.4. Available: http://beast.bio.ed.ac.uk/Tracer. Accessed 2014 Jan 21.

[54] Silvestro D, Michalak I (2011) raxmlGUI: a graphical front-end for RAxML. Org Divers Evol 12: 335–337.

[55] Shimodaira H (2002) An approximately unbiased test of phylogenetic tree selection. Syst Biol 51: 492–508. 12079646 10.1080/10635150290069913

[56] Shimodaira H (2008) Testing regions with non-smooth boundaries via multiscale bootstrap. J Stat Plan Inference 138: 1227–1241.

[57] Shimodaira H, Hasegawa M (2001) Consel: for assessing the confidence of phylogenetic tree selection. Bioinformatics 17: 1246–1247. 11751242 10.1093/bioinformatics/17.12.1246

[58] Baldwin BG, Sanderson MJ, Porter JM, Wojciechowski MF, Cambell CS, et al (1995) The ITS region of nuclear ribosomal DNA: A valuable source of evidence on angiosperm phylogeny. Ann Missouri Bot Gard 82: 247–277.

[59] Hudson RR (1990) Gene genealogies and the coalescent process. Oxford Surv Evol Biol 7: 1–44.

[60] Jang CG, Müllner AN, Greimler J (2005) Conflicting patterns of genetic and morphological variation in European *Gentianella* section *Gentianella* . Bot J Linn Soc 148: 175–187.

[61] Flagel LE, Rapp RA, Grover CE, Widrlechner MP, Hawkins J, et al (2008) Phylogenetic, morphological, and chemotaxonomic incongruence in the North American endemic genus *Echinacea* . Amer J Bot 95: 756–765. 21632401 10.3732/ajb.0800049

[62] Ramsey J, Robertson A, Husband B (2008) Rapid adaptive divergence in new world *Achillea*, an autopolyploid complex of ecological races. Evolution 62: 639–653. 18039326 10.1111/j.1558-5646.2007.00264.x

[63] Fuertes Aguilar J, Rosselló JA, Nieto Feliner G (1999) nrDNA concerted evolution in natural and artificial hybrids of *Armeria* (Plumbaginaceae). Mol Ecol 8: 1341–1346. 10447874 10.1046/j.1365-294x.1999.00690.x

[64] Fuertes Aguilar J, Rosselló JA, Nieto Feliner G (1999b) Molecular evidence for the compilospecies model of reticulate evolution in *Armeria* (Plumbaginaceae). Syst Biol 48: 735–754. 12066298 10.1080/106351599259997

[65] Baack EJ, Rieseberg LH (2007) A genomic view of introgression and hybrid speciation. Curr Opin Genet Dev 17: 513–518. 17933508 10.1016/j.gde.2007.09.001 PMC2173880

[66] Moody ML, Rieseberg LH (2012) Sorting through the chaff, nDNA gene trees for phylogenetic inference and hybrid identification of annual sunflowers (*Helianthus* sect. *Helianthus*). Molec Phylogen Evol 64: 145–155. 10.1016/j.ympev.2012.03.012 22724134

[67] Álvarez I, Wendel JF (2003) Ribosomal ITS sequences and plant phylogenetic inference. Molec Phylogen Evol 29: 417–434. 10.1016/s1055-7903(03)00208-2 14615184

[68] Kučera J, Marhold K, Lihová J (2009) *Cardamine maritima* group (Brassicaceae) in the amphi-Adriatic area: A hotspot of species diversity revealed by DNA sequences and morphological variation. Taxon 59: 148–164.

[69] Koopman WJM (2005) Phylogenetic signal in AFLP data sets. Syst Biol 54: 197–217. 16012092 10.1080/10635150590924181

[70] Ennos RA (1994) Estimating the relative rates of pollen and seed migration among plant populations. Heredity 72: 250–259.

[71] Malm JU, Prentice HC (2002) Immigration history and gene dispersal: allozyme variation in Nordic populations of the red campion, *Silene dioica* (Caryophyllaceae). Biol J Linn Soc 77: 23–34.

[72] Tyler T (2002) Geographical distribution of allozyme variation in relation to post-glacial history in *Carex digitata*, a widespread European woodland sedge. J Biogeogr 29: 919–930.

[73] Tyler T, Prentice HC, Widén B (2002) Geographic variation and dispersal history in Fennoscandian populations of two forest herbs. Pl Syst Evol 233: 47–64.

[74] Rauscher JT, Doyle JJ, Brown AHD (2002) Internal transcribed spacer repeat-specific primers and the analysis of hybridisation in the *Glycine tomentella* (Leguminosae) polyploid complex. Mol Ecol 11: 2691–2702. 12453251 10.1046/j.1365-294x.2002.01640.x

[75] Rebernig CA, Schneeweiss GM, Bardy KE, Schönswetter P, Villaseñor JL, et al (2010) Multiple Pleistocene refugia and Holocene range expansion of an abundant southwestern American desert plant species (*Melampodium leucanthum*, Asteraceae). Mol Ecol 19: 3421–3443. 20670366 10.1111/j.1365-294X.2010.04754.x

[76] Hewitt GM (2004) Genetic consequences of climatic oscillations in the Quaternary. Phil Trans R Soc B 359: 183–195. 15101575 10.1098/rstb.2003.1388 PMC1693318

[77] Médail F, Diadema K (2009) Glacial refugia influence plant diversity patterns in the Mediterranean Basin. J Biogeogr 36: 1333–1345.

[78] Kramp K, Huck S, Niketić M, Tomović G, Schmitt T (2009) Multiple glacial refugia and complex postglacial range shifts of the obligatory woodland plant species *Polygonatum verticillatum* (Convallariaceae). Pl Biol 11: 392–404. 10.1111/j.1438-8677.2008.00130.x 19470110

[79] Huck S, Büdel B, Kadereit JW, Printzen C (2009) Rangewide phylogeography of the European temperate-montane herbaceous plant *Meum athamanticum* Jacq.: Evidence for periglacial persistence. J Biogeogr 36: 1588–1599.

[80] Rešetnik I, Frajman B, Bogdanović S, Ehrendorfer F, Schönswetter P (2014) Disentangling relationships among the diploid members of the intricate genus *Knautia* (Caprifoliaceae, Dipsacoideae). Molec Phylogen Evol 74: 97ec P 10.1016/j.ympev.2014.01.028 24508604

[81] Schönswetter P, Solstad H, Garcìa PE, Elven R (2009) A combined molecular and morphological approach to the taxonomically intricate European mountain plant *Papaver alpinum* s.l. (Papaveraceae) – Taxa or informal phylogeographical groups? Taxon 17: 1326–1343.

[82] Wójcicky JJ (1991) Variability of *Prunus fruticosa* Pall. and the problem of an anthropohybridisaton. Veröff. Geobot. Inst. ETH, Stiftung Rübel, Zürich 106: 266–272.

