# Supplementary material for: Dealing with Discordant Genetic Signal Caused by Hybridisation, Incomplete Lineage Sorting and Paucity of Primary Nucleotide Homologies: A Case Study of Closely Related Members of the Genus Picris Subsection Hieracioides (Compositae)
Source: PLoS One. 2014 Sep 5;9(9):e104929. doi: 10.1371/journal.pone.0104929 (PMC4156297; doi:10.1371/journal.pone.0104929)
Supplement: Table S1 — Locality details of studied taxa, number of individuals used for the particular molecular analyses, and GenBank accession numbers. (DOC) [file pone.0104929.s001.doc]

**Table S1.** List of the populations of studied *Picris* taxa including population codes, collection data (locality, coordinates), number of individuals (*n*) used for the particular molecular analyses and the GenBank accession numbers. Seeds of the populations marked by * were obtained from the Millennium Seed Bank, Royal Botanic Gardens, Kew (Great Britain), and those marked by ** were obtained from the Seed Bank of the National Botanic Garden of Belgium, Meise (Belgium).

| **Code** | **Locality** | **Coordinates** | | **ITS**  (*n*) | **cpDNA**  (*n*) | | **GenBank acc. no.** |
| --- | --- | --- | --- | --- | --- | --- | --- |
| ***Picris hieracioides*** | | |  | | | | |
| 1 | ES, Andalusia, Pradollano, 2,370 m | 37º05´41´´N  03º23´31´´W | | 1 | 1 | KC121913, KC122119 | |
| 2 | ES, Andalusia, Capileira, 1,606 m | 36º57´42´´N  03º21´27´´W | | 1 | 1 | KC121914, KC122083 | |
| 3 | ES, Aragón, Balneario de la Panticosa, 1,576 m | 42º45´17´´N  00º14´33´´W | | 1 | 1 | KC121896, KC122069 | |
| 4 | ES, Catalonia, Vielha, 1,230 m | 42º41´11´´N  00º47´14´´W | | 1 | 1 | KC121895, KC122070 | |
| 5 | ES, Catalonia, Espot, 1,576 m | 42º33´33´´N  01º05´25´´E | | 1 | 1 | KC121909, KC122139 | |
| 6 | ES, Catalonia, Tárrega, ca 460 m | ca 41º39´00´´N  01º12´00´´E | | 2 | 2 | KC121955, KC121956, KC122137, KC122138 | |
| 7 | ES, Catalonia, between Montseny and el Brull, 740 m | 41º46´42´´N  02º23´47´´E | | 1 | 1 | KC121897, KC122136 | |
| 8 | ES, Catalonia, Llívia, 1,170 m | 42º26´38´´N  01º56´32´´E | | 1 | 1 | KC121908, KC122140 | |
| 9 | AD, Andorra, Soldeu, 1,900 m | 42º34´10´´N  01º40´41´´E | | 1 | 1 | KC121898, KC122071 | |
| 10* | GB, England, Greater London | ca. 51°30´19´´N  0°07´26´´W | | 1 | 1 | KC121954, KC122124 | |
| 11 | FR, Languedoc-Roussillon, Estavar, 1,640 m | 42º29´55´´N  02º00´49´´E | | 1 | 1 | KC121907, KC122141 | |
| 12 | FR, Franche-Comté, Bois-d’Amont, 1,102 m | 46°28´44´´N  06°05´11´´E | | 2 | 2 | KC121904, KC121905, KC122125, KC122126 | |
| 13 | FR, Provence-Alpes-Côte d’Azur, Col du Lautaret, 2,067 m | 45°02´06´´N  06°24´14´´E | | 1 | 1 | KC121937, KC122122 | |
| 14 | FR, Rhône-Alpes, la Rivine, 1,556 m | 45°04´08´´N  06°25´11´´E | | 1 | 1 | KC121899, KC122121 | |
| 15 | FR, Rhône-Alpes, Ugine, 552 m | 45°45´30´´N  06°28´18´´E | | 1 | 1 | KC121953, KC122118 | |
| 16 | FR, Franche-Comté, Les Fins, 911 m | 47°05´11´´N  06°38´21´´E | | 1 | 1 | KC121903, KC122123 | |
| 17 | FR, Franche-Comté, Pont-de-Roide, 384 m | 47°22´37´´N  06°46´00´´E | | 1 | 1 | KC121906, KC122132 | |
| 18 | FR, Alsace, Munster, 853 m | 47°54´32´´N  07°09´42´´E | | 3 | 3 | KC121800, KC121811, KC121812, KC122020, KC122134, KC122135 | |
| 19** | BE, Hainaut, Charleroi, 112 m | 50°24´50´´N  04°25´45´´E | | 1 | 1 | KC121798, KC122117 | |
| 20** | BE, Vlaams-Brabant, Wemmel, 43 m | 50°54´25´´N  04°18´13´´E | | 1 | 1 | KC121951, KC122003 | |
| 21 | IT, Piemonte, Limonetto, 1,600 m | ca 44°12´00´´N  07°34´00´´E | | 3 | 3 | KC121810, KC121935, KC121936, KC122096, KC122097, KC122098 | |
| 22 | IT, Piemonte, Breia, 799 m | 45°45´54´´N  08°18´18´´E | | 1 | 1 | KC121940, KC122100 | |
| 23 | IT, Piemonte, Gatinara, 289 m | 45°36´31´´N  08°19´26´´E | | 1 | 1 | KC121938, KC122099 | |
| 24 | IT, Trentino-Alto Adige, San Giacomo – Brentonico, 1,162 m | 45°47´58´´N  10°54´50´´E | | 1 | 1 | KC121941, KC122101 | |
| 25 | IT, Trentino-Alto Adige, Passo Piano delle Fugazze, 1,156 m | 45°45´38´´N  11°11´08´´E | | 1 | 1 | KC121939, KC122164 | |
| 26 | IT, Friuli-Venezia Giulia, Madrisio, 10 m | 45°51´41´´N  12°59´00´´E | | 1 | 1 | KC121870, KC121992 | |
| 27 | IT, Abruzzo, Prati di Tivo, 1,434 m | 42°29´57´´N  13°33´50´´E | | 1 | 1 | KC121950, KC122095 | |
| 28 | IT, Abruzzo, Valle di Selva Romana, 1,575 m | 42º07´38´´N  14º07´23´´E | | 1 | 1 | KC121949, KC122094 | |
| 29 | IT, Abruzzo, Fornace, 620 m | 42°03´56´´N  13°02´10´´E | | 2 | 2 | KC121958, KC121959, KC121989, KC121990 | |
| 30 | IT, Abruzzo, Assergi Campo, 1,236 m | 42°28´46´´N  13°21´43´´E | | 3 | 3 | KC121797, KC121960, KC121961, KC121991, KC122165, KC122166 | |
| 31 | IT, Abruzzo, L´Aquila, 421 m | 42º12´06´´N  13º24´15´´E | | 3 | 3 | KC121795, KC121796, KC121957, KC122167, KC122168, KC122169 | |
| 32 | IT, Abruzzo, Campo Felice, 1,570 m | 42°13´56´´N  13°24´39´´E | | 1 | 1 | KC121878, KC121988 | |
| 33 | IT, Basilicata, Muro Lucano, 750 m | 40°15´34´´N  15°27´25´´E | | 1 | 1 | KC121867, KC122046 | |
| 34 | IT, Calabria, Spineto Manco, 1,292 m | 39°21´24´´N  16°13´43´´E | | 1 | 1 | KC121880, KC122017 | |
| 35 | IT, Calabria, Mt. Mula, ca 1,900m | 39º45´00´´ N  16º01´00´´ E | | 1 | 1 | KC121879, KC122045 | |
| 36 | IT, Calabria, Frascineto, 453 m | 39°49´54´´N  16°15´18´´E | | 1 | 1 | KC121864, KC122013 | |
| 37 | IT, Calabria, Cosenza, 225 m | 39°10´20´´N  16°32´42´´E | | 1 | 1 | KC121874, KC122160 | |
| 38 | IT, Sicily, Palermo, Mt. Monte Cuccio, 611 m | 38º06´56´´N  13º14´33´´E | | 1 | 1 | KC121877, KC121986 | |
| 39 | IT, Sicily, Rebottone, 680 m | 38º01´43´´N  13º21´59´´E | | 1 | 1 | KC121881, KC121985 | |
| 40 | IT, Sicily, Caccamo, 612 m | 37º57´07´´N  13º43´10´´E | | 1 | 1 | KC121882, KC121987 | |
| 41 | IT, Sicily, Piano Zucchi, ca 1,100 m | ca 37º54´00´´N  13º59´03´´E | | 2 | 2 | KC121865, KC121866, KC121983, KC121984 | |
| 42 | DE, Baden-Württemberg, Titisee, 853 m | 47°54´32´´N  08°09´42´´E | | 1 | 1 | KC121901, KC122133 | |
| 43 | DE, Baden-Württemberg, Heidelberg-Schlierbach, 100 m | 49°24´28´´N  08°44´33´´E | | 2 | 2 | KC121804, KC121916, KC122116, KC122128 | |
| 44 | DE, Bayern, Graseck, 768 m | 47°28´13´´N  11°07´06´´E | | 1 | 1 | KC121915, KC122103 | |
| 45 | DE, Bayern, Fall, 780 m | 47°34´00´´N  11°31´24´´E | | 1 | 1 | KC121912, KC122115 | |
| 46 | DE, Thüringen, Wölnitz, 174 m | 50°54´18´´N  11°35´53´´E | | 1 | 1 | KC121902, KC122110 | |
| 47 | DE, Sachsen-Anhalt, Magdeburg, 84 m | 52°03´39´´N  11°35´12´´E | | 1 | 1 | KC121892, KC122148 | |
| 48 | DE, Mecklenburg-Vorpommern, Wismar, 12 m | 53°53´44´´N  11°29´13´´E | | 2 | 2 | KC121813, KC121962, KC122021, KC122022 | |
| 49 | DE, Sachsen, Leipzig, 98 m | 51°15´06´´N  12°18´33´´E | | 2 | 2 | KC121792, KC121793, KC122112, KC122147 | |
| 50 | DE, Sachsen, Pflug, 242 m | 50°59´18´´N  12°36´29´´E | | 1 | 1 | KC121884, KC122146 | |
| 51 | DE, Brandenburg, Kreis Barnim,  Barnimer Heide, leg. Royl 7045 (DE-0-B-1602800) | ca. 52°51´20´´N  13°42´27´´E | | 2 | 2 | KC121788, KC121889, KC122127, KC122149 | |
| 52 | AT, Tirol, Achenkirchen, 940 m | 47°32´11´´N  11°42´30´´E | | 1 | 1 | KC121911, KC122104 | |
| 53 | AT, Vorarlberg, Feldkirch, 550 m | 47°53´36´´N  11°50´36´´E | | 1 | 1 | KC121900, KC122180 | |
| 54 | AT, Tirol, Kitzbühel, 930 m | 47°28´12´´N  12°23´48´´E | | 1 | 1 | KC121910, KC122102 | |
| 55 | AT, Niederösterreich, Annaberg, 519 m | 47°54´59´´N  15°26´24´´E | | 1 | 1 | KC121947, KC122178 | |
| 56 | AT, Niederösterreich, Mt. Schneeberg, 543 m | 47°44´14´´N  15°44´05´´E | | 1 | 1 | KC121863, KC122048 | |
| 57 | CZ, Juhomoravský kraj, Břeclav, 166 m | 48°46´51´´N  16°54´19´´E | | 2 | 2 | KC121789, KC121790, KC122023, KC122024 | |
| 58 | CZ, Juhomoravský kraj, Rosice, 368 m | 49°11´24´´N  16°25´14´´E | | 2 | 2 | KC121791, KC121816, KC122025, KC122026 | |
| 59 | CZ, Pardubický kraj, Chrudim, 270 m | 49°57´48´´N  15°46´58´´E | | 1 | 1 | KC121888, KC122064 | |
| 60 | CZ, Úsťanský kraj, Žatec, 238 m | 50°20´44´´N  13°32´47´´E | | 1 | 1 | KC121852, KC122027 | |
| 61 | SI, Kranjska Gora, Kranjska Gora, 864 m | 46°28´19´´N  13°47´06´´E | | 1 | 1 | KC121873, KC121993 | |
| 62 | SI, Cerkno, near Cerkno, 351 m | 46°07´57´´N  13°59´48´´E | | 1 | 1 | KC121942, KC122111 | |
| 63 | SE, Skåne, Karpalund, 5 m | ca. 56°02´26´´N  14°15´32´´E | | 1 | 1 | KC121890, KC122005 | |
| 64 | SE, Öland, near Sandvik village, 7 m | ca. 57°04´36´´N  16°51´35´´E | | 1 | 1 | KC121894, KC122120 | |
| 65 | HR, Krapinsko-Zagorska županija, village of Krapina, 224 m | 46° 09´23´´N  15° 52´26´´E | | 1 | 1 | KC121868, KC122162 | |
| 66 | HR, Istarska županija, near Lupoglav, 386 m | 45°19´18´´N  14°09´25´´E | | 1 | 1 | KC121885, KC122161 | |
| 67 | HR, Primorsko-goranska županija, Cres, Vrana, 158 m | 44°48´19´´N  14°25´16´´E | | 1 | 1 | KC121869, KC122163 | |
| 68 | HR, Zadarska županija, Pirovac, 24 m | 43°49´20´´N  15°40´08´´E | | 2 | 2 | KC121875, KC121876, KC122068, KC122157 | |
| 69 | HR, Varaždinska županija, Ivanec, 232m | 46°13´14´´N  16°07´07´´E | | 1 | 1 | KC121872, KC122155 | |
| 70 | PL, Województwo zachodniopomorskie, Płocin, 21 m | 53°51´42´´N  14°33´26´´E | | 2 | 2 | KC121814, KC121815, KC122129, KC122130 | |
| 71 | PL, Województwo kujawsko-pomorskie, Šwiecie, 20 m | 53°23´20´´N  18°24´30´´E | | 1 | 1 | KC121893, KC122072 | |
| 72 | PL, Województwo kujawsko-pomorskie, Grebocin, 81 m | 53°03´38´´N  18°42´32´´E | | 1 | 1 | KC121952, KC122131 | |
| 73 | PL, Województwo śląskie, Mykanów, 236 m | 50°54´31´´N  19°12´24´´E | | 1 | 1 | KC121853, KC122084 | |
| 74 | SK, Bratislavský kraj, Záhorská Bystrica, 220 m | 48°14´05´´N  17°03´14´´E | | 1 | 1 | KC121945, KC122050 | |
| 75 | SK, Bratislavský kraj, Stupava, 224 m | 48°16´54´´N  17°03´36´´E | | 3 | 3 | KC121799, KC121801, KC121809, KC122002, KC122051, KC122052 | |
| 76 | SK, Bratislavský kraj, Bratislava-Petržalka, 132 m | 48°05´35´´N  17°05´33´´E | | 3 | 3 | KC121786, KC121787, KC121821, KC121999, KC122000, KC122018 | |
| 77 | SK, Trenčiansky kraj, Mt. Vápeč, 543 m | 48°56´29´´N  18°18´55´´E | | 1 | 1 | KC121840, KC122028 | |
| 78 | SK, Žilinský kraj, Mt. Kľak, 1,290 m | 48°58´28´´N  18°38´19´´E | | 3 | 3 | KC121778, KC121779, KC121780, KC122073, KC122074, KC122075 | |
| 79 | SK, Žilinský kraj, Lúčky, 643 m | 49°08´31´´N  19°23´03´´E | | 3 | 3 | KC121781, KC121782, KC121783, KC122085, KC122086, KC122087 | |
| 80 | SK, Žilinský kraj, Liptovský Trnovec, 588 m | 49°06´56´´N  20°20´56´´E | | 2 | 2 | KC121839, KC121871, KC122001, KC122030 | |
| 81 | SK, Žilinský kraj, Mačie Diery, 900 m | 49°15´32´´N  19°40´14´´E | | 2 | 3 | KC121784, KC121946, KC122076, KC122077, KC122078 | |
| 82 | SK, Prešovský kraj, Ždiarska dolina valley, 890 m | 49°16´06´´N  20°14´59´´E | | 2 | 2 | KC121943, KC121944, KC122044, KC122047 | |
| 83 | SK, Košický kraj, Suchá Belá gorge, 942 m | 48°56´05´´N  20°22´52´´E | | 2 | 2 | KC121794, KC121817, KC122054, KC122142 | |
| 84 | SK, Košický kraj, Geravy, 860 m | 48°52´12´´N  20°23´29´´E | | 1 | 1 | KC121785, KC122049 | |
| 85 | SK, Košický kraj, Soroška pass, 544 m | 48°37´04´´N  20°37´48´´E | | 1 | 1 | KC121841, KC122029 | |
| 86 | HU, Fejér, Rétszilas, 106 m | 46°48´44´´N  18°38´25´´E | | 2 | 2 | KC121822, KC121833, KC122033, KC122055 | |
| 87 | HU, Pest, Albertirsa, 149 m | 47°14´06´´N  19°40´02´´E | | 1 | 1 | KC121836, KC122031 | |
| 88 | HU, Komárom-Esztergom, Tata, 153 m | 47°38´08´´N  18°14´17´´E | | 1 | 1 | KC121835, KC122032 | |
| 89 | BA, Višegrad, Meremišlje, 360 m | 43°44´36´´N  19°13´17´´E | | 1 | 1 | KC121858, KC122061 | |
| 90 | RS, Južna i istočna Srbija, Korbevac, 375 m | 42º37´27´´N  22º02´32´´E | | 1 | 1 | KC121834, KC122037 | |
| 91 | RS, Šumadija i zapadna Srbija, Nova Varoš, 937 m | 43º27´34´´N  19º47´55´´E | | 1 | 1 | KC121856, KC122038 | |
| 92 | RS, Šumadija i zapadna Srbija, Suvobor, 694 m | 44°07´28´´N  20°15´56´´E | | 1 | 1 | KC121857, KC122060 | |
| 93 | ME, Podgorica, along the road E762 from Podgorica to Nikšič, 41 m | 42º27´14´´N  19º11´35´´E | | 2 | 2 | KC121819, KC121820, KC122043, KC122056 | |
| 94 | MK, Stip, Leskovica, 752 m | 41º35´33´´N  22º13´42´´E | | 1 | 1 | KC121838, KC122039 | |
| 95 | MK, Bitola, Capari, 896 m | 41º04´11´´N  21º10´33´´E | | 2 | 2 | KC121823, KC121832, KC122040, KC122041 | |
| 96 | MK, Mavrovo a Rostushe, Boletin, 762 m | 41º40´01´´N  20º36.453E | | 1 | 1 | KC121837, KC122042 | |
| 97 | GR, Makedonia Thraki, Lykodromio, 376 m | 41º12´47´´N  24º47´24´´E | | 2 | 2 | KC121824, KC121825, KC121994, KC121995 | |
| 98 | GR, Makedonia Thraki, Chamokerassa, 303 m | 41º11´47´´N  24º23´48´´E | | 2 | 2 | KC121818, KC121831, KC122151, KC122158 | |
| 99 | GR, Makedonia Thraki, Mesorachi, 230 m | 41º00´59´´N  23º50´55´´E | | 1 | 1 | KC121830, KC121996 | |
| 100 | RO, Bihor, Oradea and Chişlaz, 180 m | 47º16´08´´N  22º13´39´´E | | 2 | 2 | KC121849, KC121850, KC122035, KC122091 | |
| 101 | RO, Hunedoara, Deva, 220 m | 45º50´01´´N  22º56´20´´E | | 1 | 1 | KC121845, KC122036 | |
| 102 | RO, Hunedoara, Baru, 260 m | 45º31´56´´N  23º01´57´´E | | 1 | 1 | KC121847, KC122019 | |
| 103 | RO, Sălăj, Sălăjeni, 177 m | 47°18´46´´N  22°54´51´´E | | 2 | 2 | KC121843, KC121844, KC122034, KC122053 | |
| 104 | RO, Hunedoara, Bănita, 540 m | 45º28´23´´N  23º11´57´´E | | 3 | 3 | KC121802, KC121805, KC121803, KC122089, KC122090, KC122092 | |
| 105 | RO, Braşov, Făgăraş, 415 m | 45º49´56´´N  25º01´57´´E | | 1 | 1 | KC121846, KC122088 | |
| 106 | RO, Braşov, Braşov, 600 m | 45º39´31´´N  25º34´38´´E | | 1 | 1 | KC121917, KC122093 | |
| 107 | RO, Harghita, Hargita Băi, 1291 m | 46°22´44´´N  25°38´01´´E | | 2 | 2 | KC121806, KC121807, KC122107, KC122108 | |
| 108 | RO, Neamţ, Poiana Largului, 533 m | 47°05´28´´N  25°57´28´´E | | 1 | 2 | KC121808, KC122105, KC122106 | |
| 109 | RO, Ilfov, near Bucharest, 121 m | 44°19´04´´N  26°04´05´´E | | 1 | 1 | KC121848, KC122145 | |
| 110 | UA, Lvivska oblasť, Lisnja, 313 m | 49°25´17´´N  23°26´04´´E | | 1 | 1 | KC121948, KC122109 | |
| 111 | UA, Khmel’nyts’ka oblast’, village of Smotrych, 180 m | 48°39´02´´N  26°35´03´´E | | 1 | 1 | KC121855, KC122062 | |
| 112 | UA, Khmel’nyts’ka oblast’, village of Chrebtyev, 260 m | 48°37´33´´N  27°14´25´´E | | 1 | 1 | KC121854, KC122063 | |
| 113 | BG, Burgas, Malko Tarnovo, 541 m | 41°58´20´´N  27°29´09´´E | | 1 | 1 | KC121886, KC122152 | |
| 114 | BG, Burgas, Tzarevo, 44 m | 42°10´09´´N  27°50´16´´E | | 1 | 1 | KC121887, KC122176 | |
| 115 | BG, Burgas, Slančev Brjag, 29 m | 42°42´17´´N  27°42´31´´E | | 2 | 2 | KC121826, KC121827, KC122153, KC122156 | |
| 116 | BG, Varna, Nova Šipka, 25 m | 43°03´10´´N  27°31´39´´E | | 2 | 2 | KC121828, KC121829, KC121674, KC122174, KC122175 | |
| 117 | BG, Targovište, Razbojna, 269 m | 43°12´03´´N  26°30´42´´E | | 1 | 1 | KC121851, KC122154 | |
| 118 | BG, Veliko Tarnovo, Samovodene, 172 m | 43°08´40´´N  25°36´44´´E | | 1 | 1 | KC121842, KC122177 | |
| 119 | TR, Eskişehir, Bozuk Satih, 829 m | 39°49´08´´N  30°11´52´´E | | 1 | 1 | KC121891, KC122004 | |
| 120 | TR, Bursa, Bursa City, cca 270 m | ca. 40°11´37´´N  29°01´01´´E | | 2 | 2 | KC121859, KC121860, KC121997, KC121998 | |
| 121 | TR, Denizli, Honaz Dağ, 941 m | 37°40´25´´N  29°13´53´´E | | 1 | 1 | KC121862, KC122150 | |
| 122 | TR, Çanakkale, between Kocaçeşme and Yerlisu villages, 273 m | 40°42´27´´N  26°46´59´´E | | 1 | 1 | KC121861, KC122159 | |
| ***Picris hispidissima* (PHI)** | | |  | | | | |
| 123 | HR, Splitsko-dalmatinska županija, Omiš, 5 m | 43°27´02´´N  16°41´54´´E | | 2 | 2 | KC121925, KC121926, KC122009, KC122010 | |
| 124 | HR, Zadarska županija, Gračac, 756 m | 44°15´52´´N  16°00´32´´E | | 1 | 1 | KC121966, KC122008 | |
| 125 | HR, Dubrovačko-neretvanska županija, Dubrovnik,  145 m | 42°38´36´´N  18°07´18´´E | | 2 | 2 | KC121920, KC121921, KC122006, KC122065 | |
| 126 | HR, Istarska županija, village of Plomin, 225 m | 45°07´23´´N  14°12´08´´E | | 2 | 2 | KC121883, KC121965, KC122007, KC122173 | |
| 127 | HR, Primorsko-goranska županija, Island Krk, Omišalj, 53 m | 45°14´15´´N  14°33´24´´E | | 2 | 2 | KC121963, KC121964, KC122172, KC122179 | |
| 128 | HR, Ličko-senjska županija, Velika Paklenica valley, 30 m | 44°17´00´´N  15°27´00´´E | | 1 | 2 | KC121924, KC122113, KC122114 | |
| 129 | HR, Ličko-senjska županija, village of Karlobag, 695 m | 44°31´43´´N 15°07´32´´E | | 2 | 2 | KC121922, KC121923, KC122170, KC122171 | |
| 130 | HR, Dubrovačko-Neretvanska županija, near the village of Zaton-Doli, 124 m | 42°48´59´´N 17°46´41´´E | | 2 | 2 | KC121918, KC121919, KC122011, KC122012 | |
| 131 | ME, Danilovgrad, between the villages Kujava and Cerovo, 185 m | 42°36´27´´N  19°01´53´´E | | 2 | 2 | KC121929, KC121930, KC122057, KC122058 | |
| 132 | ME, Bar, Virpazar village, 107 m | 42°13´46´´N  19° 06´14´´E | | 2 | 2 | KC121933, KC121934, KC122016, KC122059 | |
| 133 | ME, Cetije, near Cetinje village, 485 m | 42°24´29´´N  18°46´45´´E | | 2 | 2 | KC121927, KC121928, KC122066, KC122067 | |
| 134 | ME, Bar, Bukovnik, 614 m | 42°13´16´´N  18°57´58´´E | | 2 | 2 | KC121931, KC121932, KC122014, KC122015 | |
| ***Picris olympica* (PO)** | | |  | | | | |
| 135 | TR, Bursa, Uludağ Mts., 2059 m | 40°05´35´´N  29°07´52´´E | | 2 | 2 | KC121967, KC121968, KC122079, KC122080 | |
| 136 | TR, Kütahya, Saphanedağ Mts., 1817 m | 39°02´41´´N  29°17´06´´E | | 2 | 2 | KC121969, KC121970, KC122081, KC122082 | |
| 137 | TR, Izmir, Boz Dağlari Mts., 1677 m | 38°19´50´´N  28°06´29´´E | | 2 | 2 | KC121971, KC121972, KC122143, KC122144 | |
| ***Picris japonica* (PJ)** | | |  | | | | |
| 138 | JP, Akita pref., Kitaakita-gun, Tashiro-cho, Hirataki, 339 m  339 m | 40º22´23´´N  140º26´20´´E | | 2 | 2 | KC121973, KC121974, KC122183, KC122184 | |
| 139 | JP, Hokkaido, Atsuta-gun, Atsuta-mura, Morai, 66 m | 43º21´01´´N  141º29´25´´E | | 2 | 2 | KC121975, KC121976, KC122181, KC122182 | |
| ***Picris nuristanica* (PN)** | | |  | | | | |
| 140 | KG, Tian-Schan, Fergana (Fergana Kyrka Toosu), 2,800 m | ca. 43º23´11´´N  71º47´11´´E | | 3 | 3 | KC121977, KC121978, KC121979, KC122185, KC122186, KC122187 | |
| ***Picris capuligera* (PCA)** | | |  | | | | |
| 141 | MA, S of Fez, 890 m | 33º48´12´´N  04º59´19´´W | | 1 | 1 | KJ417459, KJ417470 | |
| ***Picris galilea*  (PGa)** | | |  | | | | |
| 142* | JO, Irbid, 586 m | ca. 32º32´44´´N  35º51´26´´E | | 2 | 2 | KJ417460, KJ417461, KJ417471, KJ417472 | |
| ***Picris pauciflora* (PPa)** | | |  | | | | |
| 143 | GR, Crete, Xania, Theodoros, ca. 70 m | 35º30´39´´N  24º02´ 05´´E | | 1 | 1 | KJ417462, KJ417473 | |
| 144 | TR, Karaislah village, 859 m | 40º01´55´´N   29º07´33´´E | | 1 | 1 | KJ417463, KJ417474 | |
| ***Picris rhagodioloides* (PRA)** | | |  | | | | |
| 145 | GR, Crete, Xania, Kokkinochorio, ca. 200 m | 41º27´21´´N  24º14´05´´ E | | 1 | 1 | KJ417464, KJ417475 | |
| 146 | IL, Golan Hights, Ein Zivan village, 1,024 m | 33º06´30´´N  35º48´03´´E | | 1 | 1 | KJ417465, KJ417476 | |
| ***Picris scaberrima* (PSc)** | | |  | | | | |
| 147 | IT, Castrovillari, 386 m | 39º49´17´´N  16º12´11´´E | | 1 | 1 | KJ417468, KJ417479 | |
| ***Picris sinuata* (PSi)** | | |  | | | | |
| 141* | TN, Sousse | ca. 35°50´01´´N  10°38´02´´E | | 2 | 2 | KC121980, KC121981, KC122188, KC122189 | |
| ***Picris strigosa* (PSt)** | | |  | | | | |
| 149 | IR, Kalibar, 2,171m | 38º 56´47´´N  46º59´12´´ E | | 1 | 1 | KJ417466, KJ417477 | |
| 150 | TR, Denizli, Honazdağ, 1,449 m | 37º39´31´´ N  29º15´33´´ E | | 1 | 1 | KJ417467, KJ417478 | |
| ***Helminthotheca aculeata* (HA)** | | |  | | | | |
| 151 | IT, Sicily, Custonaci, 120 -200 m, leg. M. A. Ortiz et K. Tremetsberger, 8.6.2008, herb. WU | 38º06´05´´ N  12º39´59´´ E | | 1 | 1 | KJ417469, KJ417480 | |
| ***Helminthotheca echioides* (HE)** | | |  | | | | |
| 152 | IT, Sicily, Rebottone, 680 m | 38º01´43´´N  13º21´59´´E | | 1 | 1 | KC121982, KC122190 | |
